# Insecticidal and Nematicidal Contributions of Mexican Flora in the Search for Safer Biopesticides

**DOI:** 10.3390/molecules24050897

**Published:** 2019-03-04

**Authors:** Beatriz Hernández-Carlos, Marcela Gamboa-Angulo

**Affiliations:** 1 Instituto de Agroindustrias, Universidad Tecnológica de la Mixteca, Huajuapan de León, Oaxaca 69000, Mexico; bhcarlos@mixteco.utm.mx; 2 Unidad de Biotecnología, Centro de Investigación Científica de Yucatán, Calle 43 No. 130, Col. Chuburná, Mérida 97200, Mexico

**Keywords:** asteraceae, *Haemonchus*, insecticides, *Meloidogyne*, mexican plants, nematicides, pesticides, plant extracts, *Spodoptera*

## Abstract

Plant metabolites have been used for many years to control pests in animals and to protect crops. Here, we reviewed the available literature, looking for the species of Mexican flora for which extracts and metabolites have shown activity against pest insects and parasitic nematodes of agricultural importance, as well as against nematodes that parasitize domestic cattle. From 1996 to 2018, the search for novel and eco-friendly biopesticides has resulted in the identification of 114 species belonging to 36 botanical families of Mexican plants with reported biological effects on 20 insect species and seven nematode species. Most plant species with detected pesticide properties belong to the families Asteraceae, Fabaceae, and Lamiaceae. Eighty-six metabolites have been identified as pesticidal active principles, and most have been terpenoids. Therefore, the continuation and intensification of this area of research is very important to contribute to the generation of new products that will provide alternatives to conventional pesticide agents. In addition, future studies will contribute to the recognition and dissemination of the importance of propagating plant species for their conservation and sustainable use.

## 1. Introduction

Pest control in the agricultural sector requires a greater number of alternative products that meet food safety, sustainability, and environmental care requirements. One of the strategies used to obtain new natural agents for protecting crops and domestic animals is the exploration of a diversity of plants and their metabolites [1,2]. Natural products with pesticidal properties have been demonstrated to be an important source of compounds which are used as raw materials in the development of new protective agents, both in their natural form or as semisynthetic derivatives exhibiting better effects. In addition, the chemical structures of the active components of natural products have guided the synthesis of other active compounds [3]. The exploration and use of natural products are currently increasing, with a greater focus on identifying metabolites for use in the treatment of human diseases, including parasistism and plant diseases, as well as products for use in pest control in the agricultural sector [4,5,6,7,8,9].

The biotic wealth of Mexico, which includes large tropical zones, is widely recognized as being among the greatest in the world, with Mexico harbouring an estimated 23,314 species of native vascular plants, approximately 49.8% of which are endemic [10]. Nevertheless, the amount of biodiversity prospecting for natural products in Mexico is low, and as in other countries, it has primarily focused on the search for products to control diseases or plagues that affect humans [11,12,13]. With respect to agricultural applications, most studies have focused on identifying antimicrobial agents rather than insecticides, nematicides, and herbicides [5,13,14,15]. Regarding plants with insecticidal properties, the results of previous studies have identified 24 Mexican plant species with pesticidal potentials that are used in different regions of the country, many of which have been identified as medicinal plants by ethnobotanical antecedents [16]. In contrast, few botanical prospecting studies have been performed to identify plants with activities against phyto and zoonematode pests. Worldwide, few plant extracts have been shown to have an acaricidal activity, three of which are from Mexican flora and were tested on *Rhipicephalus microplus*, and only seven pure natural compounds have been identified as active principles [17]. Undoubtedly, *Tagetes erecta* (Asteraceae), a native plant of Mesoamerica, is currently recognized as one of the most promising plant species given its diverse biological activities against human and plant pathogens as well as against multiple pests [9,18].

Therefore, this work reviews the Mexican flora with extracts or secondary metabolites that have shown biological activity against pest insects or parasitic nematodes. Some plant species that were introduced to Mexico, such as *Allium sativum*, *Azadirachta indica*, and *Ricinus communis*, among others, are also discussed. The information was compiled from all of the electronic databases available at the institution, which included Google Scholar, SciFinder, PubMed, Redylac, Scopus, and Science Direct, among others.

## 2. Insecticidal Compounds and Plant Extracts

Research on natural products for controlling pest insects that affect plants has led to the identification of 85 plant species with extracts and metabolites that are effective against at least one of the evaluated targets. These plants belong to 26 botanical families, predominantly Asteraceae (31%), Lamiaceae (14%), Meliaceae (7%), Annonaceae (6%), Chenopodiaceae (6%), Fabaceae (5%), and Rutaceae (5%), with the rest belonging to the families Acanthaceae, Anacardiaceae, Asparagaceae, Bignoniaceae, Brassicaceae, Burseraceae, Cactaceae, Caricaceae, Convolvulaceae, Euphorbiaceae, Lauraceae, Magnoliaceae, Papaveraceae, Petiveraceae, Piperaceae, Phytolaccaceae, Poaceae, Solanaceae, and Verbenaceae (<5% each).

Twenty pest insects were evaluated in the reviewed studies. The maize pest *Spodoptera frugiperda* J.E. Smith (Lepidoptera: Noctuidae) is the most frequently tested target together with *Spodoptera littoralis* Boisduval (Lepidoptera: Noctuidae) and *Spodoptera exigua* Hübner (Lepidoptera: Noctuidae), collectively representing 30% of the target pests assayed in the reviewed studies, and these species were followed by *Sitophilus zeamais* Motschulsky (Coleoptera: Curculionidae, 14%), the sucker *Bemisia tabaci* Gennadius (Homoptera: Aleyrodidae, 11%), and *Trialeurodes vaporariorum* West. (Homoptera: Aleyrodidae, 7%). The remaining targets included *Anastrepha ludens* Loew (Diptera: Tephritidae), *Bactericera cockerelli* (Hemiptera: Psylloidea), *Copitarsia decolora* Guenée (Lepidoptera: Noctuidae), *Dactylopius opuntiae* Cockerell (Hemiptera: Coccoidea), *Leptinotarsa decemlineata* Say (Coleoptera: Chrysomelidae), *Prostephanus truncatus* Horn (Coleoptera: Bostrichidae), *Scyphophorus acupunctatus* Gyllenhaal (Coleoptera: Curculionidae), *Stomoxys calcitrans* Linneo (Diptera: Muscidae), *Tenebrio molitor* Linnaeus (Coleoptera: Tenebrionidae), *Trichoplusia ni* Hübner (Lepidoptera: Noctuidae), and *Zabrotes subfasciatus* Boheman (Coleoptera: Bruchidae). Other targets assayed included *Aedes aegypti* Linnaeus, *Anopheles albimanus* C.R.G. Wiedemann, and *Culex quinquefasciatus* Say (Diptera: Culicidae), which have been included in this review because they are all very important pest insects of humans and are also virus vectors.

In this review, first, the insecticidal compounds isolated and identified in enriched fractions (as alkaloids and terpenes) and essential oils (EOs) from Mexican plants are described by the targeted pests. The second part includes plant extracts which the active principles of are not yet known.

### 2.1. Spodoptera *sp.*

During investigations carried out on the control of *Spodoptera* sp. (*S. frugiperda* and *S. littoralis*), 43 effective natural compounds have been identified including terpenes (**1**–**30**), flavonoids (**31**–**35**), stilbenes (**36**–**38**), a coumarin (**39**), a ketone (**40**), and fatty acids (**41**–**44**). In addition, enriched fractions with metabolites that were identified as alkaloids (**45**–**50**) have been described. All of these compounds were isolated from 21 plant species and exhibited different degrees of effectiveness against the assayed pest insects, with the most active metabolites obtained from plants of the Asteraceae family (*Cedrela dugessi*, *Cedrela salvadorensis*, *Gutierrezia microcephala*, *Parthenium argentatum*, and *Roldana barba-johannis*), the Fabaceae family (*Lupinus aschenbornii*, *Lupinus montanus*, and *Lupinus stipulates*), and the Asparagaceae family (*Yucca periculosa*), which induced the strongest median lethal concentration (LC_50_ ≤ 65 ppm) against *S. frugiperda*. Other plant species with minor activities against *S. frugiperda* included *Carica papaya*, *Crescentia alata*, *Lippia graveolens*, *Myrtillocactus geometrizans*, *Ricinus communis*, *Ruta graveolens*, *Vitex Hemsley*, and *Vitex mollis*. In addition, five species from the genus *Salvia* and a member of the family Asteraceae (*Senecio toluccanus*) were found to have active compounds against *S. littoralis*.

#### 2.1.1. Terpenes

The tocotrienols and hydroquinones isolated from the methanol extract (MEx) of the aerial parts of *R. barba-johannis* (Asteraceae) included sargachromenol (**1**), methyl and acetyl sargachromenol derivatives (**2**, **3**), sargahydroquinoic acid (**4**), methyl and acetyl sargahydroquinoic acid derivatives (**5**, **6**), and sargaquinoic acid (**7**). Metabolites **1**, **3**, and **6** showed potent insecticidal activity against the fifth-stage larvae of *S. frugiperda*, with median lethal dose (LD_50_) values of 2.94, 3.89, and 4.83 ppm, respectively. Metabolite **4** was most effective against first-instar *S. frugiperda* larvae, with a LC_50_ of 5.77 ppm. Furthermore, acetylated metabolite **3** was the most potent compound against the emergence of *S. frugiperda* adults from pupae, while the efficacy was further increased using a mixture of acetylated compounds **1**, **3**, and **7** (LD_50_ = 3.26 ppm) [19]. Furthermore, Cespedes [20] identified two cycloarten-type triterpenes, argentatin A (**8**) and argentatin B (**9**), from a methanol extract (MEx) of the aerial parts of *P. argentatum*. Although both metabolites showed good insecticidal and growth inhibition activities, the MEx was consistently more potent than either triterpene alone. Methanol extract, **8**, and **9** showed a potent toxicity towards *S. frugiperda* adults, with LD_50_ values of 3.1, 12.4, and 19.8 ppm, respectively. In addition, the insecticidal activities of MEx and compound **8** against the fifth-instar larvae of *S. frugiperda* were tested, with LC_50_ values of 6.4 and 17.8 ppm and median mortality concentration (MC_50_) values of 6.9 and 21.3 ppm, respectively. In agreement with these results, the observed growth and relative growth indices seven days after treatment with both metabolites and MEx revealed a delay in the time of *S. frugiperda* pupation and adult emergence and an increase in deformities. Acetylcholinesterase inhibition (83.5% and 100%) was observed using MEx at 5 and 25 ppm, respectively, but not for the pure compounds (90–100% at 50 ppm).

The *G. microcephala* clerodane diterpene bacchabolivic acid (**10**) and its synthetic methyl ester (**10a**) were shown to cause significant mortality (MC_50_ = 10.7 and 3.46 ppm, respectively) towards *S. frugiperda* neonatal larvae, good toxicity against adults (LD_50_ = 6.59 and 15.05 ppm, respectively), and moderate acetylcholinesterase inhibitory activity [21]. The leaves of two Meliaceae species, *C. salvadorensis* and *C. dugessi*, were shown to produce a mixture of photogedunin α and β (**11**, **12**) and gedunin (**13**). The mixture of compounds **11** and **12**, as well as **13** and its acetate derivative (**13a**), caused good *S. frugiperda* larval mortality (LC_50_ = 10, 8, and 39 ppm, respectively) [22]. A labdane-type anticopalic acid (**14**) from *Vitex hemsleyi* showed an effective antifeedant dose of 90.6 ppm against sixth-instar *S. frugiperda* larvae [23]. Sterols isolated from the aerial parts of *M. geometrizans* (Cactaceae), including macdougallin (**15**), peniocerol (**16**), and a mixture of the two metabolites **15**:**16** (4:6), displayed a high toxicity towards *S. frugiperda* (LD_95_ = 285, 125, and 135 ppm, respectively). In addition, at 20 ppm, the mixture of **15** and **16** drastically resulted in the total inhibition of *S. frugiperda* pupation and the emergence of adults [24].

Terpenes with noticeable activity against *S. frugiperda* (100 ppm: 65–80% larval mortality) have been identified in enriched fractions from *Crescentia alata*, including ningpogenin (**17**), β-sitosterol (**18**), stigmasterol (**19**), and 6β,7β,8α,10-tetra-*p*-hydroxybenzoyl-*cis*-2-oxabicycle-(4.3.0)nonan-3-one (**20**) [25,26]. Guevara [27] reported that monoterpenes thymol (**21**) and carvacrol (**22**) were the major components in a hexanic extract of *L. graveolens* leaves. This extract caused deformations in *S. frugiperda* adults at different concentrations (10–100 ppm).

**Figure 1:**
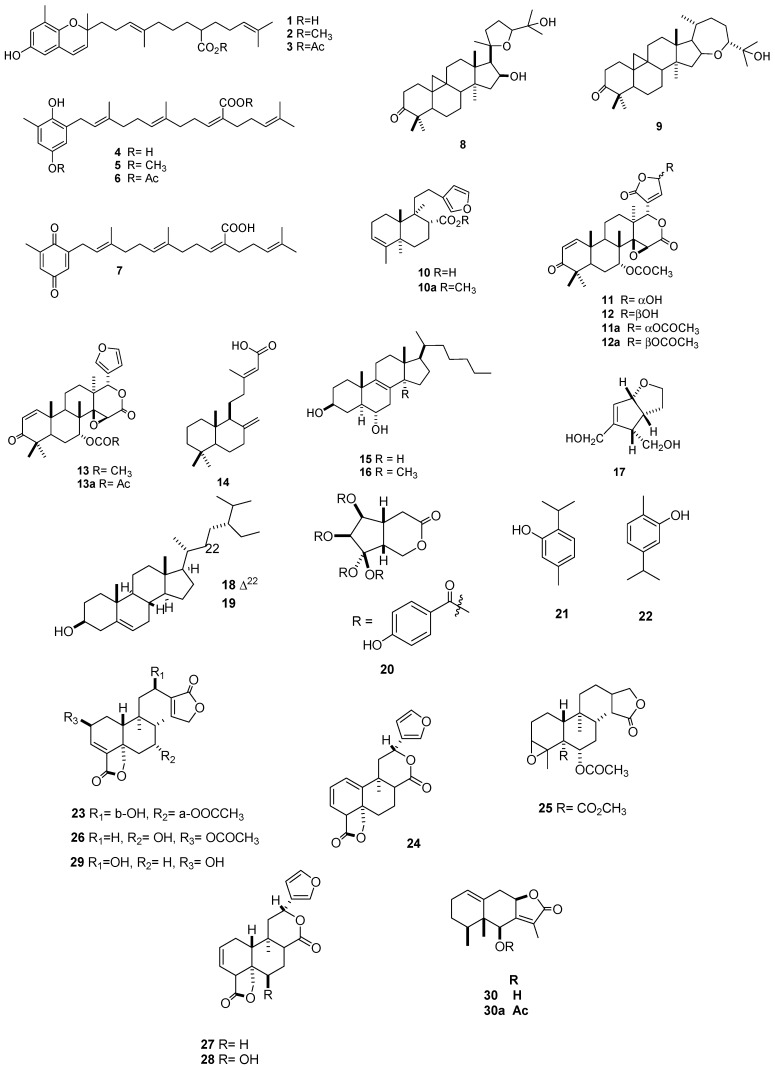
Terpenes with activity on *Spodoptera* sp.

The pest *S. littoralis* was also shown to be sensitive to seven antifeedant clerodane-type diterpenoids obtained from several *Salvia* species (AI_50_ < 90 ppm). These diterpenoids included kerlinolide (**23**); 1(10)-dehydrosalviarin (**24**) from *Salvia lineata*; from *Salvia keerlii*, 13,14-dihydro-3,4 epoxy-melissodoric acid methyl ester acetate (**25**), 2β-acetoxy-7α-hydroxy-*neo*-clerodan-3,13-dien-18,19:16.15-diolide (**26**) from *Salvia melissodora*; salviarin (**27**) from *Salvia rhyacophila*; and 6β-hydroxysalviarin (**28**) and semiatrin (**29**) from *Salvia semiatrata*. The most effective of these was **25**, with an AI_50_ value of 1 ppm [28]. The metabolite toluccanolide A (**30**), isolated from *S. toluccanus*, and its acetate derivative (**30a**) showed a significant antifeedant effect against *S. littoralis* (57% and 69.6%, respectively) after an application of this compound (50 µg/cm^2^) to leaves (Table 1, Figure 1) [29].

#### 2.1.2. Flavonoids

**Figure 2:**
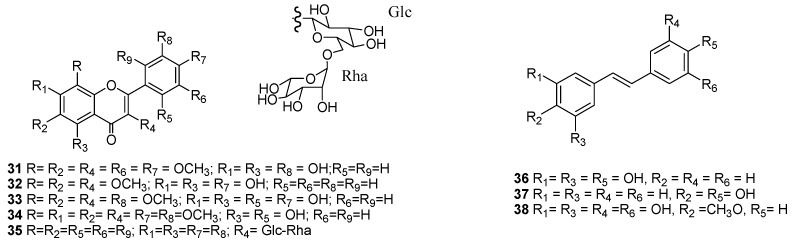
Insecticidal flavonoids (**31**–**35**) and stilbenes (**36**–**38**) effective on *Spodoptera frugiperda*.

Flavonoids isolated from the aerial parts of *G. microcephala* exhibited moderate effects against *S. frugiperda*, with these compounds including 5,7,2′-trihydroxy-3,6,8,4′,5′-pentamethoxyflavone (**31**), 5,7,4′-trihydroxy-3,6,8-trimethoxyflavone (**32**), 5,7,2′,4′-tetrahydroxy-3,6,8,5′-tetramethoxyflavone (**33**), and 5,2′-dihyhydroxy-3,6,7,8,4′,5′-hexamethoxyflavone (**34**). Flavone **31** displayed the lowest LC_50_ value (3.9 ppm) against neonatal *S. frugiperda* larvae [21]. In addition, flavones **31**–**34** exhibited 93.7–100% acetylcholinesterase inhibitory activity at 50 ppm (Table 2, Figure 2).

Rutin (**35**) is a flavonol glycoside-reported *R. graveolens* constituent (Figure 2), which was also tested and showed no effect towards *S. frugiperda* [30].

#### 2.1.3. Stilbenes

Stilbenes identified from the bark of *Y. periculosa* (Asparagaceae) included resveratrol (**36**), 4,4′-dihydroxystilbene (**37**), and 3,3′,5,5′-tetrahydroxy-4-methoxystilbene (**38**), with **38** being the most potent and exhibiting an LC_50_ value of 5.4 ppm towards neonatal larvae at seven days and a median growth inhibition (GI_50_) value of 3.45 ppm at 21 days (Table 3, Figure 2) [31].

#### 2.1.4. Coumarin and Ketone

The leaves of *R. graveolens* were shown to produce psoralen (**39**) and a median chain ketone 2-undecanone (**40**), both of which were effective against neonatal *S. frugiperda* larvae. However, metabolite **39** was more potent than **40**, with larval mortalities of 100% and 50% respectively observed at a concentration of 1 mg/mL (Table 4) [30].

#### 2.1.5. Fatty Acids

Additional compounds with reported activity against *S. frugiperda* include palmitic (**41**), oleic (**42**), linoleic (**43**), and linolenic (**44**) acids (Table 5), which exhibited LV_50_ values of ≤ 1354 ppm, with the most active compounds being unsaturated fatty acids. These active fatty acids were detected in *C. papaya* seeds and *R. communis* leaves grown in Mexico [32,33]. Both of these plant species are widely distributed, and *R. communis* is recognized for its pesticidal effects and high fatty acid content [34]. Furthermore, the powdered seed of *C. papaya* has been shown to cause larval mortality and weight reduction in *S. frugiperda* [35,36].

#### 2.1.6. Alkaloidal Fractions

**Figure 3:**
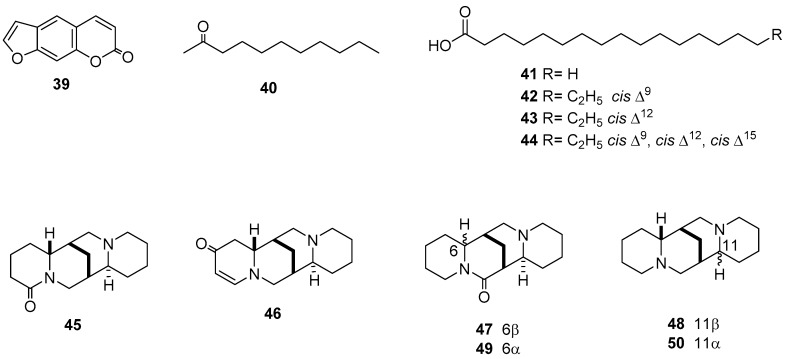
Metabolites with activity on *Spodoptera frugiperda*.

Alkaloid-enriched fractions from leaves of three species of *Lupinus* (Fabaceae) showed remarkable toxic effects against *S. frugiperda* (LD_50_ = 16–70 ppm). These fractions primarily contained lupanine (**45**), multiflorine (**46**), sparteine (**47**), aphylline (**48**), α-sparteine (**49**), and *epi*-aphylline (**50**) (Table 6, Figure 3), with a commercial standard of **47** used during the evaluations. Interestingly, *L. montanus* and *L. aschenbornii* had high amounts of **47** (640 and 780 μg/g, respectively), whereas it was absent from *L. stipulatus*, which instead contained **48** and **50** as major alkaloids (280 and 307 μg/g, respectively). The alkaloidal fraction of *L. stipulatus* was the most toxic and fast-acting against *S. frugiperda*, with an LD_50_ value of 20 μg/mL at seven days, similar to that observed for **47** (LD_50_ = 11 μg/mL) [37].

#### 2.1.7. Plant Extracts with Activity against *Spodoptera* sp.

The crude organic extracts of 10 plant species exhibited effective insecticidal activities against *S. frugiperda,* with one showing activity against *S. exigua*, the results of which are shown in Table 7. These plants included *Bursera copallifera*, *Bursera grandiflora*, *Bursera lancifolia*, *Ipomoea murucoides*, *Ipomoea pauciflora*, *Salvia connivens*, *Salvia microphylla*, *Tagetes erecta*, *Trichilia havanensis*, and *Vitex mollis*.

Against *S. exigua*, only the activity of an extract from *T. havanensis* seeds was reported, with an acetonic extract and its supernatant oil causing significant larval mortality and weight reduction. Furthermore, the acetone extract caused a noticeable delay in the development of *S. exigua* larvae when used at 500 mg/L [38].

The insecticidal activity of *V. mollis* extracts (dichloromethane, chloroform-methanol, and methanol) towards *S. frugiperda* was very interesting. A chloroform-methanol (1:1) extract from *V. mollis* leaves caused noteworthy mortality against *S. frugiperda* larvae, with an LC_50_ value of 13.63 ppm observed, greater than that of previously reported terpenes (vide infra). In addition, the percentage of larvae reaching pupation decreased in the presence of all of the extracts [39]. As expected, leaf and flower extracts of *T. erecta* showed activity against *S. frugiperda* larvae. At 500 ppm, the acetonic extract from leaves was the most effective, with a 50% reduction in larval weight observed after seven days. However, the hexane, acetone, and ethanol leaf extracts all exhibited lethal activities against *S. frugiperda* larvae, with observed LC_50_ values of 312.2, 246.9, and 152.2 ppm, respectively [40].

Other organic plant extracts with activity against *S. frugiperda* include acetonic extracts of *B. copallifera*, ethyl acetate extracts of *B. lancifolia*, and a methanol extract of *B. grandifolia*, which caused deformations in pupae or adults at different concentrations; acetylcholinesterase is also inhibited by these extracts [41,42]. In addition, *I. murucoides*, *I. pauciflora*, *S. connivens*, and *S. microphylla* extracts displayed slight effects against first-stage larvae of *S. frugiperda* at high concentrations (Table 7) [42,43,44,45].

### 2.2. Aedes aegypti, Anopheles albimanus, and Culex quinquefasciatus

**Figure 4:**
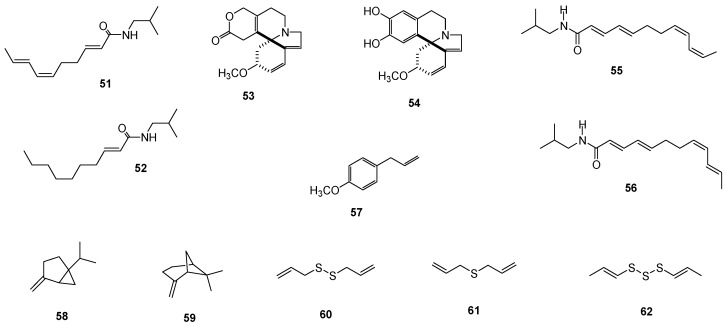
Metabolites with effect on *Aedes aegypti*, *Anopheles albimanus*, and *Culex quinquefasciatus*.

The extracts and metabolites of 11 plant species displayed activity against the Culicides *A. aegypti*, *A. albimanus*, and *C. quinquefasciatus*, vectors of the human diseases, dengue fever, malaria, and lymphatic filariasis, respectively. These plant species included *A. indica*, *Argemone mexicana*, *Erythrina Americana, Heliopsis longipes*, *Persea americana*, *Pseudocalymma alliaceum*, *Pseudosmodingium perniciosum*, *Ruta chalepensis*, *Salmea scandens*, *Thymus vulgaris*, and *Zanthoxylum fagara* (Table 8, Table 9 and Table 10 and Figure 4).

#### 2.2.1. Alkaloids

An alkamide named affinin (**51**), isolated from *H. longipes* roots, and its reduced product *N*-isobutyl-2*E*-decenamide (**52**) were moderately active against *A. aegypti* (LC_50_ = 7.38 and 36.97 mg/L, respectively). Moreover, the Coleoptera *A. albimanus* was more sensitive to these compounds, with LC_50_ values of 4.24 and 7.47 mg/L, respectively. However, a crude ethanol extract displayed lower lethal activity against the larval stage of *A. albimanus* and *A. aegypti*, with LC_50_ values of 2.48 and 4.07 mg/L, respectively (Table 8) [46]. The alkaloidal fraction from *E. americana* seeds induced high *C. quinquefasciatus* larval mortality, with an LC_50_ value of 87.5 mg/L. After chromatographic purification, β-eritroidina (**53**) and erisovina (**54**) were obtained and tested; however, these pure compounds exhibited lower *C. quinquefasciatus* larvicidal activities in comparison with the alkaloidal fraction (LC_50_ = 225 and 399 mg/L, respectively) [47]. In contrast, EOs from *S. scandens*’ stem bark caused a potent lethal effect on *A. albimanus* larvae (2.5 µg/mL), with the isomers *N*-isobutyl-(2*E*,4*E*,8*Z*,10*Z*)-dodecatetraenamide and *N*-isobutyl-(2*E*,4*E*,8*Z*,10*E*)-dodecatetraenamide (**55**, **56**; 39.7%) constituting the majority of the compounds in this EO [48].

#### 2.2.2. EOs

Among the assayed EOs, the EO obtained from leaves of *S. scandens* was the most active and had the lowest LC_50_ of 0.3 µg/mL on the larvae of *A. aegypti* [48]. *Culex quinquefasciatus* larvae were moderately sensitive to EOs from the leaves of *P. americana* (800 mg/L: 57.5% mortality) and *P. alliaceum* (LC_50_ = 385.29 ppm). The EO from *P. americana* was observed to contain estragole (**57**, 61.86%), sabinene (**58**, 15.16%), and α-pinene (**59**, 14.26%), while that of *P. alliaceum* consists primarily of diallyl disulphide (**60**, 50.05%), diallyl sulphide (**61**, 11.77%) and trisulphide di-2-propenyl (**62**, 10.37%) (Table 8, Figure 4) [49,50].

#### 2.2.3. Plant Extracts

The screening of extracts from six plants for activity against the fourth-instar *A. aegypti* larvae identified those of *A. mexicana* and *P. perniciosum* as the most effective (Table 9). Hexane and acetone extracts from *A. mexicana* seeds and hexane extracts from the bark of *P*. *perniciosum* showed the lowest larvicidal activities, with LC_50_ values of, 80, 50, and 20 µg/mL, respectively [51]. Other organic extracts observed to have larvicidal activity against *A. aegypti* include those of *R. chalepensis*, *T. vulgaris*, and *Z. fagara*, exhibiting notable LC_50_ values of 1.8, 4.4 and 75.1 µg/mL, respectively [52]. In contrast, the aqueous extract of *A. indica* showed slight effects towards four different instars of *C. quinquefasciatus* (LD_50_ = 410–550 ppm) [53].

### 2.3. Anastrepha ludens

Foliarn and stem extracts from three species of the family Annonaceae, *Annona diversifolia*, *A. lutescens*, and *A. muricata*, as well as one species of the family Magnoliaceae, *Magnolia dealbata*, showed good activity against the Mexican fruit fly *A. ludens* (Coleoptera). Among the assayed extracts, the aqueous extracts from stems exhibited the best effect at 100 μg/mL, with the greatest effect (95.9%) caused by *A. lutescens* (Table 10) [54,55].

### 2.4. Bactericera Cockerelli

The potato psyllid (*B. cockerelli*) displayed sensitivity to hexanol extracts of *A. muricata* seeds, with a lethal effect observed using 193.5 ppm after 72 h (Table 10) [56].

### 2.5. Bemisia tabaci

To date, five studies have reported on the use of natural Mexican plant products in whitefly (*B. tabaci*) management. The results of these studies identified 11 Mexican plants with extracts that are effective against various *B. tabaci* life stages (eggs, nymphs, and adults). The plant species included *Acalypha gaumeri*, *Agave tequilana*, *Annona squamosa*, *A. indica*, *Capsicum chinense*, *Carlowrightia myriantha*, *C. ambrosioides*, *Petiveria alliacea*, *Piper nigrum*, *Pluchea sericea*, and *Trichilia arborea*.

#### Plant Extracts

Cruz-Estrada [57] investigated the effects of extracts from six plant species against *B. tabaci* eggs and reported that aqueous extracts from the leaves of *A. gaumeri*, *A. squamosa*, *P. alliacea*, and *T. arborea* exhibited activity (LC_50_ = 0.36–0.42%, *w*/*v*), as did the ethanol extracts of *P. alliacea* (LC_50_ = 2.09 mg/mL) and *T. arborea* (LC_50_ = 2.14 mg/mL). The latter two species showed the highest activity against *B. tabaci* nymphs (LC_50_ = 1.27 and 1.61 mg/mL, respectively). In parallel, leaf extracts from *A. indica* plants grown in Mexico were assayed. The toxic effects of the aqueous extracts of native plants were similar to those of *A. indica* aqueous extracts (LC_50_ = 0.30%, *w*/*v*) and were greater than those of the *A. indica* ethanolic extract against eggs (LC_50_ = 3.60 mg/mL) and nymphs (LC_50_ = 2.57 mg/mL). *A. tequilana* juice (undiluted) and its hexanic extract (2%) promoted *B. tabaci* nymph mortality (100% and 91%, respectively), which is interesting given the significant quantities of juice obtained from the waste of this agave (Table 11) [58].

In another study (Table 11), the ethanol extracts of mature *C. chinense* fruits (creole orange variety) showed slight repellency and mortality effects against *B. tabaci* adults (LC_50_ = 29.4% *w*/*v*, LT_50_ = 7.31 h). The concentration of capsaicinoids in the fruit of the habanero pepper was 1193.6 mg/kg. Capsaicinoids have been reported to have toxic and repellent effects against insects [59]. Ethanolic extracts from the leaves of *C. ambrosioides* and the fruits of *P. nigrum* showed good lethal activity against *B. tabaci*, with the lowest LC_50_ of 1.6% (*w*/*v*) observed for the *P. nigrum* extracts. Furthermore, *P. nigrum* produces high ethanolic extract yields (3.69%), and this plant is inexpensive and accessible [60]. Finally, *P. sericea* is an interesting Asteraceae species which the extracts of have been shown to be effective against *B. tabaci* adults, with acetone, aqueous, and ethanolic extracts of the leaves shown to have moderate repellence activity (RI_50_ of 0.52–0.78) [61].

### 2.6. Copitarsia Decolora and Dactylopius Opuntiae

The EOs of *Beta vulgaris*, *C. graveolens*, and *Chenopodium berlandieri* subsp. *nuttalliae* reduced the fecundity and fertility (75–99%) of *C. decolora* and increased (19–38%) the lengths of the larval and pupal periods (Table 12) [62].

**Figure 5:**
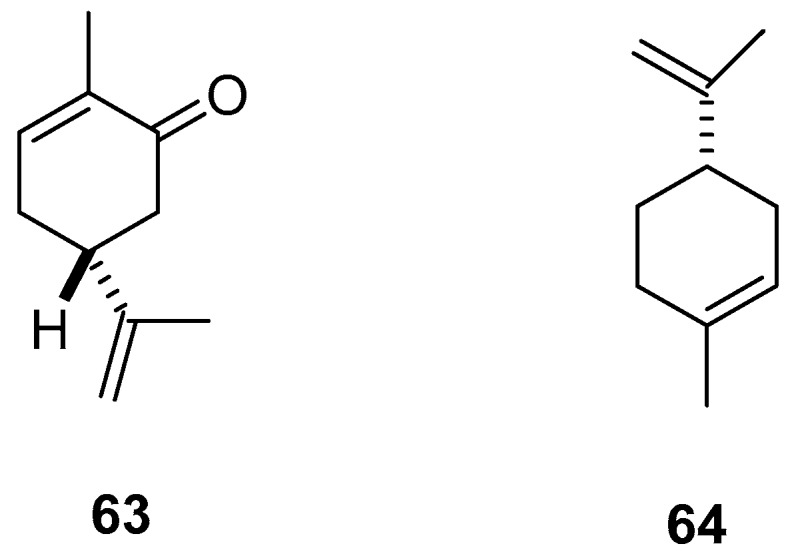
The majority components in the EOs of *Mentha spicata* effective on *Dactylopius opuntiae.*

Vazquez-García [63] reported that EOs obtained from *Cymbopogon winterianus*, *L. graveolens*, *Mentha spicata*, and *Ocimum basilicum* were active against the first-instar larvae of the prickly pear cochineal *D. opuntiae*, with LC_50_ values ranging from 0.8–6.6 mL/100 mL. The most effective was the EO of *M. spicata*, the primary constituents of which were carvone (**63**, 61.03%) and limonene (**64**, 15.18%) (Table 12, Figure 5).

### 2.7. Leptinotarsa decemlineata

**Figure 6:**
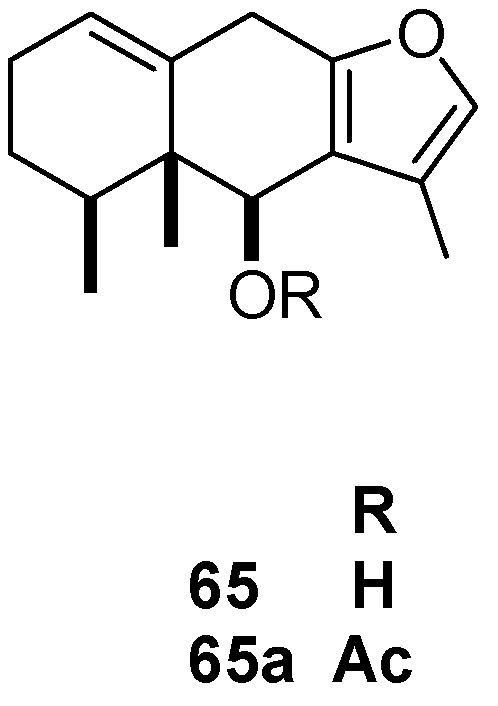
Insecticidal metabolite 6-hydroxyeuryopsin from *Senecio toluccans*.

The metabolite 6-hydroxyeuryopsin (**65**) isolated from *S. toluccanus*, and its acetate derivative (**65a**) exhibited a higher antifeedant effect (85 and 93.3% at 50 μg/cm^2^, respectively) against the Colorado potato beetle (*L. decemlineata*) than did *S. frugiperda* (*vide supra*) (Table 13, Figure 6) [29].

### 2.8. Prostephanus truncates

**Figure 7:**
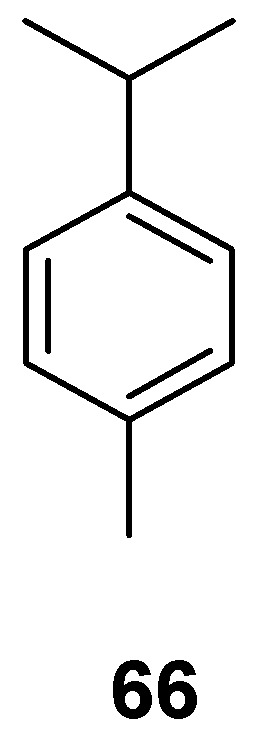
Majority metabolite (*p*-Cimene) from extract of *Lippia palmeri*.

The larger grain borer (*P. truncates*) was shown to be susceptible to EO from the leaves of *Lippia palmeri*, with an LC_50_ value of 320.5 μL/L observed after 72 h. After the application of the EOs, a strong repellency against the insect at 200 μL/L was observed, and no insect emerged at 500 μL/L in 24 h. These EOs primarily contained **22** (58.9%) and *p*-cimene (**66**, 21.8%) as majority compounds (Table 14, Figure 7) [64].

### 2.9. Sitophilus zeamais

The EOs of 14 plant species with activities against the stored grain pest *S. zeamais* were compiled. These EOs were primarily derived from members of the Asteraceae family (*Aster subulatus*, *Bahia absinthifolia*, *Chrysactinia mexicana*, *Erigeron longipes*, *Eupatorium glabratum*, *Heliopsis annua*, *Heterotheca inuloides*, *Hippocratea celastroides*, *Hippocratea excelsa*, *Senecio flaccidus*, *Stevia serrata*, and *Zaluzania peruviana*) as well as members of the Rutaceae and Verbenaceae families (*Stauranthus perforates* and *L. palmeri*, respectively).

#### 2.9.1. Terpenes

**Figure 8:**
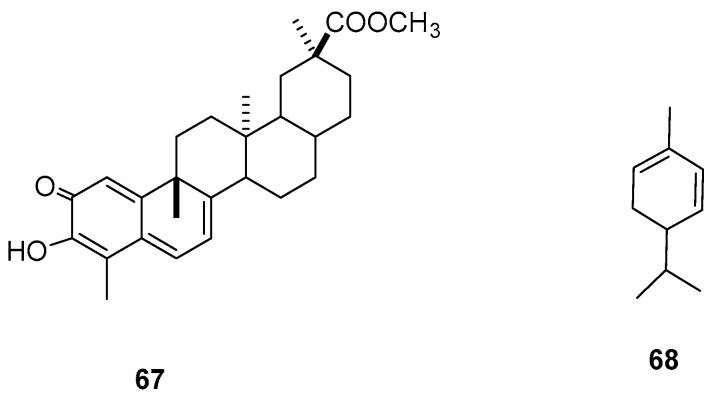
Metabolites with activity against *Sitophilus zeamais.*

The triterpenoid pristimerin (**67**) was isolated from the roots of *H. excelsa* and displayed a high antifeeding activity index (AAI) of 89% and slight mortality (M = 16%) when used in a 1% formulation against *S*. *zeamais* (Table 15, Figure 8) [65].

#### 2.9.2. EOs

A bioactive EO from *E. glabratum* exhibited high activity against female and male *S. zeamais*, with LC_50_ values of 16 and 20 µL/mL, respectively, and median lethal times of 53 and 70 h, respectively. Chromatographic analyses of *E. glabratum* EO revealed the presence of α-pinene (**59**) and α-phellandrene (**68**, 19.6%) as the major compounds (29.5%) [66]. In contrast, the pest insect *S. zeamais* exhibited a slight sensitivity to EO from *L. palmeri* leaves, with LC_50_ value of 441.45 μL/L against adults after 48 h. In addition, this EO induced total repellency against maize weevil adults, with no emergence observed using a concentration of 1000 μL/L after 24 h, with major EO components having been previously described (**21** and **66**) (Table 15, Figure 8) [64].

#### 2.9.3. Plant Extracts

Juárez-Flores [67] screened flower powder and leaf powders from 81 plant species belonging to the Asteraceae family. Among the 162 plant powders tested (1%, *w*/*w*), twelve powders showed remarkable lethal activities (>80%) against *S. zeamais*, but only two inhibited adult emergence (<22 insects), *B. absinthifolia* and *C. Mexicana* (Table 15). The most effective of these powders were those produced from the leaves of *C. mexicana*, which caused a mortality of 98% and no adult emergence. Similarly, the root powder of *S. perforates* mixed with maize kernel (3%) displayed total mortality against *S. zeamais* [68], while an acetone extract produced from the roots of *H. celastroides* and its precipitate resulted in slight antifeeding activity index values of 72.3 and 73.8 against the stored grain pest *S. zeamais*, respectively (Table 15) [65].

### 2.10. Stomoxys calcitrans and Scyphophorus acupunctatus

**Figure 9:**
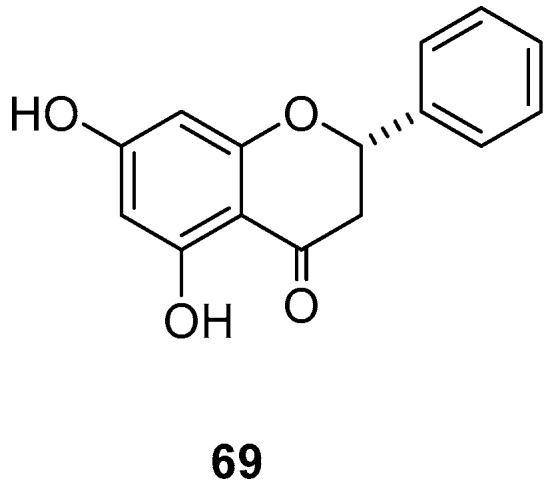
Metabolite effective on *Stomoxys calcitrans.*

The flavanone pinocembrine (**69**) obtained from the aerial parts of *Teloxys graveolens* showed an LC_50_ value of 418.69 μg/mL against the third-stage larvae of the stable fly *S*. *calcitrans*, an ectoparasite of mammals (Table 16, Figure 9) [69] 

Valdés-Estrada [70] reported that seed powders (15%) from *Trichilia havanensis*, *C. papaya*, and *Annona cherimola* had good effects (100, 90, and 63%, respectively) on the mortality of the larvae of *S. acupunctatus*. All powders inhibited the weight of the agave weevil. The most effective was A. *cherimola*. (Table 16).

### 2.11. Tenebrio molitor and Trichoplusia ni

Sterols **15** and **16** (Figure 1) from *M. geometrizans* (Cactaceae) and their combination (6:4) exhibited a high toxicity against the last-instar larvae of *T. molitor*, the yellow mealworm, causing acute toxicities with 5, 3, and 0% survival at 100 ppm, respectively. Interestingly, **15**, **16**, and their combination induced shortened *T. molitor* pupation and emergence, and many of the pupae died (Table 17) [24].

Only one report described assays against the cabbage looper *T. ni*, where volatile organic compounds from *A. indica* stems promoted significant neonatal and larval mortality (24 and 77%, respectively) at 1 g doses and an LD_50_ of 5.6 g after 7 days (Table 17) [71].

### 2.12. Trialeurodes vaporariorum

In reviewing investigations on the effectiveness of Mexican plant products against the greenhouse whitefly, the species *Arundo donax*, *Petiveria alliacea*, *Phytolacca icosandra*, *Piper auritum*, *Raphanus raphanistrum*, and *Tagetes filifolia* were compiled.

#### 2.12.1. EOs

**Figure 10:**
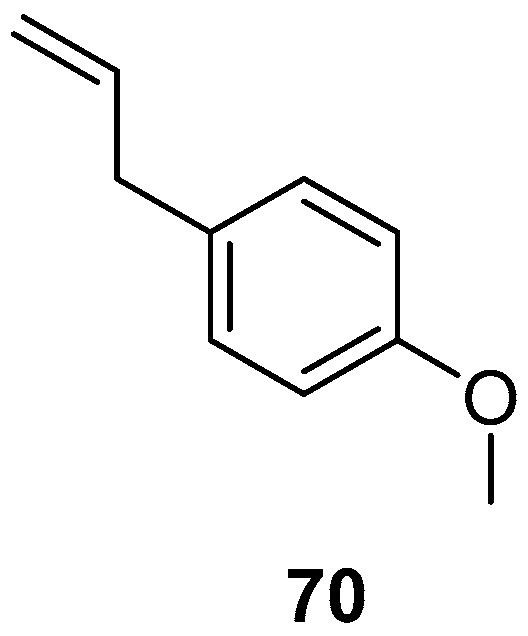
Metabolites active against *Sitophilus zeamais.*

Native populations of *T. filifola* in Mexico contain high proportions of anethole, a phenylpropene present in the EOs from the plant. Therefore, the EOs from the flowers, leaves, and whole plants of *T. filifolia* were tested together with a commercial standard of *trans*-anethole (**70**) against *T. vaporariorum*. The lowest LC_50_ value was observed using **70** (Figure 10), which produced an LC_50_ value of 1.74 mg/mL and a median oviposition inhibition concentration (IOC_50_) of 1.55 mg/mL, followed by the floral oil (LC_50_ = 6.59 mg/mL), the foliar oil (LC_50_ = 10.29 mg/mL), and the whole plant oil (LC_50_ = 9.99 mg/mL). Another parameter measured was the median repellent concentration (RC_50_), with the floral oil being the most effective with an RC_50_ value of 0.13 mg/mL against *T. vaporariorum*. The second instar of the nymphal stage of *T. vaporariorum* was noticeably sensitive to foliar oil (Table 18) [72].

#### 2.12.2. Plant Extracts

Mendoza-García [73] reported that an ethanolic extract of *P. auritum* was the most toxic extract (LC_50_ = 116 mg/mL) tested against *T. vaporariorum* and that an aqueous extract of *R. raphanistrum* effectively inhibited oviposition (IOC_50_ = 77.3 mg/mL) against the greenhouse whitefly.

Evaluations of extracts applied to tomato crops under greenhouse conditions were reported to control *T. vaporariorum*. In one study, aqueous, methanol, and dichloromethane extracts from *P. alliacea* leaves showed remarkable LC_50_ values of 16.6, 13.3, and 3.5%, respectively [74]. In contrast, methanolic extracts from *A. donax* and *P. icosandra* exhibited slightly higher target LC_50_ values of 34.79 and 36.47%, respectively, under greenhouse conditions (Table 18) [75].

### 2.13. Zabrotes subfasciatus

The species *L. palmeri* and *Senecio salignus* exhibited effective activities against *Z. subfasciatus*, the main pest of common beans (*Phaseolus vulgaris*). A 0.07% solution of a root powder of the Asteraceae species *S. salignus* exerted lethal toxicity by contact against bean weevil adults after five days. When the concentration was increased, fewer days were required to control the pest, with a 0.07% solution producing LC_50_ values of 0.03% and 0.08% after 3 days and median lethal times of 1.21 and 3.20 days observed for male and females, respectively. Therefore, males were more sensitive than females. In addition, the authors determined the optimal size of the root powder that should be used (<0.25 mm particles) [76].

#### EOs

EOs obtained from leaves of *L. palmeri* collected in the localities of Puerto de Oregano (PO) and Alamo (Al) exhibited lethal and ovicidal activities against *Z. subfasciatus* at 1.35 µL/g, with two months of persistence. EOs from leaves collected in PO was slightly more lethal than EOs obtained from leaves collected in Al. A comparison of the components of the two EOs revealed a number of differences, with carvacrol (**22**, 37.35%), thymol (**21**, 24.56%), and *p*-cimene (**64**, 15.62%) being abundant in EO from PO, whereas **64** (33.7%) and **22** (18.32%) were abundant in EOs from Al (Table 19) [77].

## 3. Nematicidal Compounds and Plant Extracts

To date, very few bioprospecting studies have been performed to identify plants with nematicide effects. In this review, we identified reports describing 37 plant species with toxic activities towards plant and animal nematode parasites. These plant species belong to 21 botanical families, with those of the family Fabaceae (41%) being predominant. A total of 18 secondary metabolites were identified as active principles or presenting an active fraction against at least one of the parasitic nematodes tested in the reviewed studies, including terpenes (**71**–**82**), flavonoids (**44**, **69**, **83**, and **86**), a pehnylpropaoid (**84**), and a coumarin (**85**). These metabolites were obtained from *C. anuum*, *Gliricidia sepium*, *Leucaena leucocephala*, *Microsechium helleri, Sicyos bulbosus*, and *T. graveolens*.

### 3.1. Plant Extracts Effective against Parasitic Plant Nematodes

Although data on the subject is scarce, we focused on compiling reports on plants that have toxic effects on phytonematodes *Meloidogyne incognita*, *Meloidogyne javanica*, and *Nacobbus aberrans*. A total of twelve metabolites from *M. helleri*, *S. bulbosus*, and *C. annuum* have been purified and identified as active principles against plant parasite nematodes.

#### 3.1.1. *Meloidogyne javanica*

**Figure 11:**
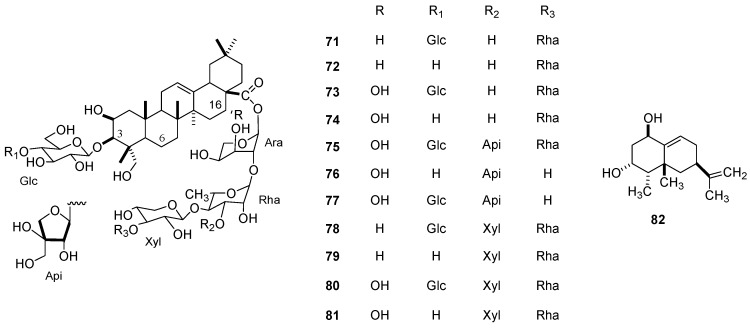
Metabolites effective against *Meloidogyne javanica* and *Nacobbus aberrans*.

Seven saponins isolated from *S. bulbosus*, namely, tacacoside B3 (**71**) and C (**72**),16-OH tacacoside B3 (**73**), durantanin III (**74**), heteropappus saponin 7 rhamnoside (**75**), and heteropappus saponin 5 and 7 (**76**–**77**), were the active compounds responsible for the nematicidal effect against *M. javanica* J_2_ (73.8–100% mortality at 0.5 µg/µL). Highly similar compounds, such as amole F-G (**78**, **79**) and 16-OH amole F-G (**80**, **81**), were isolated from *M. helleri* and caused lower (<8%) J_2_ immobility at the 0.5 µg/µL dose [78]. In addition, the hexane extract from the leaves of *L. graveolens* caused significant mortality against *M. javanica* J_2_ with an LC_50_ of 0.672 mg/mL (Table 20, Figure 11). [27].

#### 3.1.2. *Nacobbus aberrans*

The capsidiol (**82**) produced by *C. annuum* (Solanaceae) was reported to affect *N. aberrans* (Table 19). Pure capsidiol caused an 80% immobility in the J_2_ of *N. aberrans* after exposure for 72 h at a concentration of 1 μg/mL and caused a nematostatic effect (Table 20, Figure 11) [79].

#### 3.1.3. *Meloidogyne incognita*

Plant extracts from *Calea urticifolia*, *E. winzerlingii*, and *Tephrosia cinerea* were shown to have lethal activities against *M. incognita* (Table 20). An aqueous extract from the roots of *C. urticifolia* was tested on second-stage *M. incognita* juveniles under greenhouse conditions. The results showed that 50% (*w*/*v*) of the *C. urticifolia* root extract effectively reduced gall formation (50%) and the number of eggs (72% reduction) on tomato seedlings that had been inoculated with 1000 eggs and 130 *M. incognita* J_2_ [80]. Ethanol extracts from the roots of *C. urticifolia*, the stems of *T*. *cinerea*, and the leaves of *E. winzerlingii* produced immobility in *M. incognita* J_2_ (>80%) when applied at 250 ppm. Finally, the ethanol extract from *E. winzerlingii* leaves was very active against *M. incognita* and had the lowest LC_50_ (133.4 ppm) of the tested extracts [81].

### 3.2. Plant Extracts with Activity against Parasitic Animal Nematodes

To date, 27 plant species have been identified with an effect against animal nematodes, 12 of which belong to the family Fabaceae (43%). The relevant studies primarily focused on the control of *Haemonchus contortus* (93%): one study investigated *Haemonchus placei*, and three investigated *Trichostrongylus colubriformis*, zooparasites of sheep. In addition, three studies focused on *Cooperia puntacta* and *Cyatostomin* sp., zooparasites of grazing cattle and horses, respectively, and one focused on *Ascaridia galli*, a bird parasite. Herein, the active plant extracts are included, as well as some fractions or subfractions, with the predominant compounds described by the authors. Only five natural compounds were reported to have an anthelmintic activity against animal nematodes, two of which were purified and identified from plant species and the remaining two as enriched fractions, with compound rutin (**35**) assayed as a commercial standard.

#### 3.2.1. *Ascaridia galli*

Only one study investigated the effect of metabolites from *T. graveolens* (Amaranthaceae) against *A. galli*. Flavonoid **69** (Figure 9) was the active ingredient isolated from the aerial parts of *T. graveolens*, and it had an LC_50_ of 623.5 μg/mL against *A. galli* (Table 21) [69].

#### 3.2.2. *Cooperia puntacta*

Plant species with ovicidal activity against *C. puntacta* included *G. sepium* and *L. leucocephala.* These plants were extracted with water, acetone–water 30:70, and acetone solvents, and all of these fractions were tested. For each plant, at least one of the extracts showed ovicidal activity. The most effective were the acetone extract from *G. sepium* and the aqueous extract from *L. leucocephala*, which showed significant LC_50_ values of 1.03 and 7.93 mg/mL on egg hatching inhibition (EHI), respectively. The addition of a tannin inhibitor (polyethylene glycol) in all of the extracts showed that, with the exception of the *G. sepium* acetone extract, all exhibited enhanced ovicidal effects. Next, an aqueous extract of *L. leucocephala* was fractionated using chromatographic methods. Among the fractions obtained, the highest ovicidal effect was observed in LlC1F3, with an LC_50_ value of 0.06 mg/mL detected on *Cooperia* spp. The analytical data indicated that the majority of components in LlC1F3 were quercetin (**83**, 82.21%) and caffeic acid (**84**, 13.42%) [82,83].

**Figure 12:**
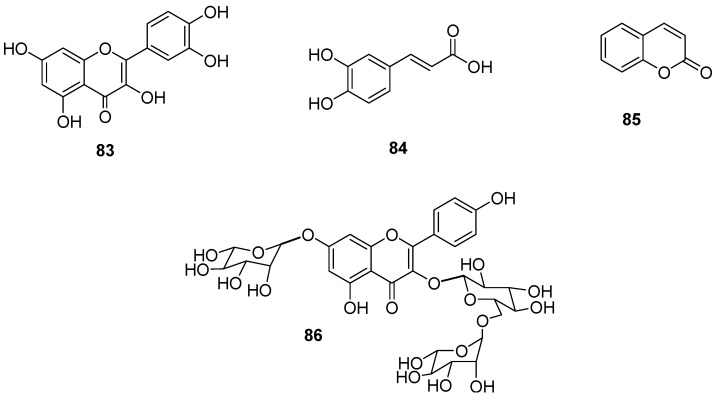
Metabolites from *Gliricida sepium* and *Leucaena leucocephala* with activity on *Cooperia* sp.

In contrast, the metabolite 2H-chromen-2-one (**85**) was purified from the acetone extract of *G. sepium* by bio-guided fractionation. Metabolite **85** had the highest ovicidal effect (EC_50_ of 0.024 mg/mL), EHI, and embryonic development against *C. puntacta* [84]. A second metabolite isolated from the leaves of *G. sepium* was identified as oxytroside (**86**) which inhibited the *C. punctata* exsheathment process at 2400 µg/mL (Table 21, Figure 12) [85].

#### 3.2.3. *Cyatostomin* sp.

An investigation on the control of the zooparasitic nematode *Cyatostomin* sp. using plant extracts was recently reported [86]. The authors indicated that methanol extracts from the leaves and bark of *Diospyros anisandra* (Ebenaceae) and the leaves and stems of *P. alliacea*, which were collected in the rainy seasons, showed promising activities in controlling the eggs and the development of L_1_ *Cyastotomin* sp. larvae. The highest ovicidal activity was produced by the bark extract of *D. anisandra*, followed by the leaf extract, both of which were collected in the rainy season. These extracts presented LC_50_ values of 10.28 and 18.48 µg/mL on the EHI, respectively, while extracts from *P. alliacea* exhibited lower lethal activities (LC_50_ ≥ of 28.27 µg/mL). However, *P. alliacea* stems, which were also collected in the rainy season, induced the failed eclosion of larvae (90.7% at 75 µg/mL). The continued study of both plant species was highly recommended (Table 21) [86].

#### 3.2.4. *Haemonchus* sp.

##### *Haemonchus* *placei*

A hydroalcoholic extract with significant activity against *H. placei*, was obtained from *Caesalpinia coriaria*. In this case, the extracts from fruits presented a greater activity than the leaves, with LC_50_ values of 3.91 and 11.68 mg/mL, respectively [87].

##### *Haemonchus* *contortus*

In ruminants, *H. contortus* is one of the most important gastrointestinal parasitic nematodes in sheep and goats, as well as *H. placei*, a hematophagous parasite in bovines. Several plant extracts exhibited promising activities in controlling the larval stage of *H. contortus in vitro* (Table 22. Among these extracts, the dichloromethane extract from *Phytolaccca icosandra* leaves (Phytolaccaceae) was one of the most active, with an LD_50_ of 0.90 mg/mL on larval migration inhibition and an LD_50_ of 0.28 mg/mL on egg hatch inhibition (EHI) in *H. contortus*. Additionally, ethanolic extracts from the same plant caused >92% of EHI at a 0.9 mg/mL *in vitro* level [88]. In addition, the methanolic extract from *Gliricidia sepium* (Fabaceae) displayed a good EHI effect, with an ED_50_ value of 394.96 µg/mL [89]. The hydroalcoholic extract from the leaves of *Acacia cochliacantha* (Fabaceae) showed total mortality against eggs of *H. contortus*. However, this extract was used at a high concentration (100 mg/mL), and its organic fraction obtained with ethyl acetate displayed one of the lowest EHI at an LC_50_ of 0.33 mg/mL. This EHI effect increased ten-fold when it was subfractionated with dichloromethane to produce soluble and precipitate subfractions, with the low LC_50_ values of 0.06 and 0.04 mg/mL observed, respectively. The ethyl acetate fraction was enriched with caffeoyl and coumaroyl derivatives [90]. The hydroalcoholic extract from *C. coriaria* showed a slightly higher effect against *H. contortus* larvae than on *H. placei*. In this case, the extracts from fruits presented LC_50_ values of 1.63 and 3.98 mg/mL, respectively [87]. In addition, the ethanol extract from the seeds of *C. papaya* (Caricaceae) induced an EHI of 92% at 2.5 mg/mL [91].

The extracts of partially purified tannins obtained from the leaves of *Arachis pintoi*, *L. leucocephala*, *Guazuma ulmifolia*, and *Manihot esculenta* reduced the migration of the third-stage larvae of *H. contortus* by 69.9–87.4% at 4.5 µg/mL and 74.2–100% at 45 µg/mL after 96 h of exposure. However, an ovicidal effect from these plants was not observed [92]. Alonso-Diaz [93] confirmed the role of tannins in the larvicidal effect of *L. leucocephala* and other tropical Fabaceae, *Acacia pennatula* and *Lysiloma latisiliquum*, with larval migration inhibitions (LMI) of 51–53.6% at 1200 µg/mL through the use of polyvinyl polypyrrolidine, an inhibitor of tannins. In contrast, *Piscidia piscipula* was not affected. Vargas-Magaña [94] demonstrated that tannins in a 30% acetone–water extract (3600 µg/mL PBS) from the leaves of *Laguncularia racemose* blocked the eclosion of eggs of *H. contortus* (50.29%). Besides, *Senegalia gaumeri* induced an EC_50_ of 401.8 and 83.1 µg/mL of EHI and larval mortality on *H. contortus,* respectively [95].

In *in vitro* studies, other investigations reported a lesser effect (20–40 mg/mL) on *H. contortus* larval mortality, including the hexane extract from the aerial parts of *Prosopis laevigata*, an acetone extract from the stem of *B. copallifera* [96], a hydro-methanolic extract from *Larrea tridentata* and aqueous extracts from *Cydista aequinoctialis*, *Heliotropium indicum*, and *Momordica charantia* (Table 22) [97,98].

There are seven reports on *in vivo* experiments that describe the effects of plant extracts. One of these studies included a mixture of extracts from the bulbs of *A. sativum* and the flowers of *T. erecta*. First, the extracts alone or in combination were tested *in vitro*. After 72 h, the lowest larval mortality of *H. contortus* (L_3_) occurred at an LC_50_ of 1.3 mg/mL, which was induced by the mixed extract (Table 22). Subsequently, it was administered in one dose of 100 µg/mL (40 mg/mL) to gerbils infected with *H. contortus* (L_3_). After 13 days, the nematode in the gastric lumen of both treatment and control animals were counted. The highest larvae population reduction (LPR) was 87.5%, which was induced by the *T. erecta* and *A. sativum* mixed extracts. Each extract of these plants alone showed a lower effect in comparison with their combination in both assays, suggesting a synergistic action [99]. Similarly, Zamilpa [100] reported that a combined extract from the aerial parts of *Castela tortuosa* and *C. ambrosioides* induced a 57.36% population reduction on L_3_ *H. contortus* in infected gerbils (Table 23). In contrast, *in vitro*, the lowest lethal activity was produced by a hexane extract of *C. ambrosioides* (LC_50_ = 1.5 mg/mL) at 72 h (Table 22). Other hexane extracts administered (100 µg/mL at 40 mg/mL) to gerbils was from *Prosopis laevigata*, which reduced parasite population (42.5%) [101].

**Figure 13:**
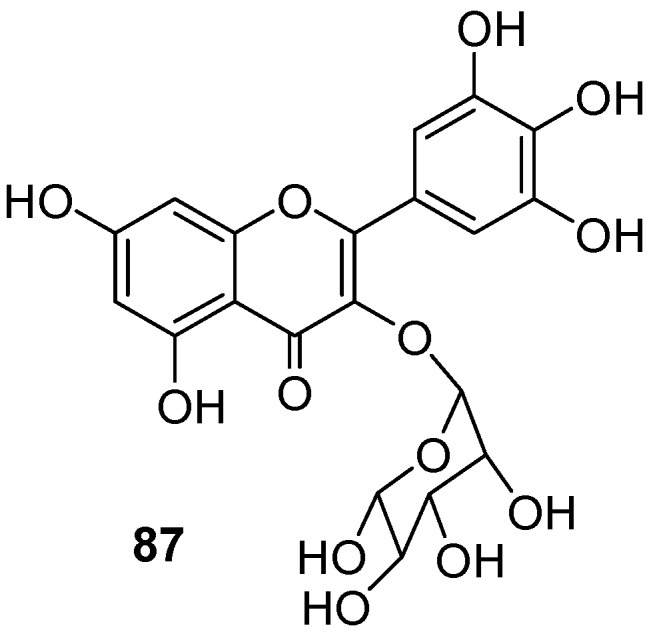
Majority component (Myricitrin) of active extract from *Lysiloma acapulcensis* eaves.

An organic ethyl acetate fraction obtained from aqueous extracts of *Lysiloma acapulcensis* leaves showed a high EHI on L_3_ (94.85%) at 6.25 g/mL and a 100% larval mortality at 50 mg/mL after 72 h at the *in vitro* level. Subsequently, an organic fraction of dry and ground leaves of *L. acapulcensis* and the flavonol rutin (**35**) used to treat infected sheep were tested *in vivo*. The reduction in the excretion of eggs per gram (EPGR) of faeces was recorded, with **35** and the ethyl acetate fraction exhibiting a 66.2 and 62.9% EPGR at a concentration of 10 and 25 mg/kg body weight (BW), respectively. The application of the ethyl acetate fraction was more effective than dried leaves (5 g/kg BW), presenting a 62.9% EPGR. The chromatographic separation of the ethyl acetate fraction revealed the presence of the flavonol myricitrin (**87**) as a major component, though this enriched fraction was not tested (Figure 13). In this experiment, the larvae of *Cooperia curticei*, *H. contortus*, and *Teladorsagia circumcincta* and the eggs of *Trichuris* sp. from faeces were identified by morphological and morphometric analyses [102]. Another *in vivo* test was reported with the ethanolic extract from *P. icosandra* leaves which was encapsuled and orally administered to infected goats. Results showed a reduction of 72% in *H. contortus* eggs/g of faeces at two doses of 250 mg/kg BW, on day 11 post-treatment (Table 23). Fatty acids and a ketone were detected in the ethanol extract of *P. icosandra* as major components [103].

In further studies, a hydroalcoholic extract from *Oxalis tetraphylla* (Oxalidaceae) leaves was orally applied daily (20 mg/kg BW) for eight days to lambs infected with *H. contortus.* The results showed a 45.6% reduction in the number of eggs/gram of faeces. Flavonol compounds in *O. tetraphylla* were also detected [104].

Finally, an *in vivo* test in goats, Creole male kids, experimentally infected with L_3_ *H. contortus* was reported. In this investigation, kids were fed fresh leaves (10% of the total diet) of *A. cochliacantha*, *G. ulmifolia*, and *Pithecellobium dulce* (Fabaceae) for sixty days. A lower EPG was observed in kids fed with *A. cochliacantha* and *P. dulce*, with 1.28 Log^10^ and 1.48 Log^10^, respectively. Moreover, the total body weight in kids noticeably increased with *P. dulce* foliage in the diet, with 0.2% (control) to 2.4% kg/animal (treatment) weight gained, which was attributed to the decrease in parasite load [105] (Table 23).

#### 3.2.5. *Trichostongyus colubriformis*

With regards to the nematode *T. colubriformis*, the extracts from three species of the family Fabaceae (1200 ppm), *Acacia pennatula*, *L. leucocephala*, and *Lysiloma latisiliquum*, reduced the migration of *T. colubriformis* third-stage larvae by 71%, 72%, and 56%, respectively (Table 24) [106].

## 4. Conclusions

**Figure 14:**
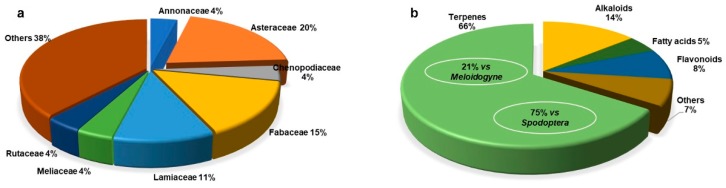
The percentage of (**a**) plant families explored and (**b**) types of metabolites isolated from native plants of México that are active on some parasitic pest.

This review demonstrates the relevant pesticidal activity of several native plant species of Mexico, the majority of which were reported at the *in vitro* level, while some were reported in *in vivo* assays. Unfortunately, at present, research on bioprospecting plant species from Mexican flora with the aim of developing natural pesticides against insects and nematode pests is still in its early stages. To date, only 114 species of Mexican plants with biological activity against insects or nematode pests have been reported, most of which belong to the Asteraceae (20%), Fabaceae (15%), and Lamiaceae (11%) families (Figure 14). The investigations on the activities of these plants have primarily focused on evaluating the biological activity of raw vegetable extracts or their enriched fractions, and less than 35% have led to the purification, identification, and evaluation of the active compounds. Among the most common metabolites with activity detected against some of the tested targets are terpenes (58%), followed by phenols and flavonoids. A mixture of extracts or their pure compounds provides a strategy in the search for natural and safer pesticides. Despite these limitations, species with a high potential for effectiveness were identified for further study in the development of biotechnological products.

Evaluations of promising plant extracts in the field are needed to identify appropriate formulations. Therefore, the use of an adequate and low-cost extract should be considered during *in vitro* evaluations. Although botanical pesticides are less persistent in the environment, toxicological studies on beneficial organisms and mammals should still be performed.

The high diversity of plant species in Mexico coupled with the increasing demand and urgency for new natural pesticides makes it extremely important to continue bioprospecting studies in this country. Additional studies will help generate new and alternative natural products that can improve the biological effectiveness, lower residuals, and increase the innocuousness of agricultural products as well as decrease their presence in foods. These studies will contribute to the recognition and dissemination of the importance of propagating plant species for their conservation and sustainable use.

## Figures and Tables

**Table 1:** Insecticidal terpenes from Mexican flora effective on *Spodoptera* sp.

Insect	Species/Family	Plant Part	Compound/Extract (Toxicity)	Ref.
*S. frugiperda*	*Roldana barba-johannis* * Asteraceae	AP	Sargachromenol (**1**) (LD_50_ = 2.94 ppm on fifth instar, 24 h; LC_50_ = 19.12 ppm on first instar, 7 days) Methyl sargachromenol (**2**) (LD_50_ = 15.52 ppm on fifth instar, 24 h; LC_50_ = 20.76 on first instar, 7 days) Acetyl sargachromenol (**3**) (LD_50_ = 3.89 ppm on fifth instar, 24 h; LC_50_ = 33.31 ppm on first instar, 7 days) Sargahydroquinoic acid (**4**) (LD_50_ = 10.17 ppm on fifth instar, 24 h; LC_50_ = 5.77 on first instar, 7 days) Methyl sargahydroquinoic acid (**5**) (LD_50_ = 14.89 ppm on fifth instar, 24 h; LC_50_ = 62.02 on first instar, 7 days) Acetyl sargahydroquinoic acid (**6**) (LD_50_ = 4.83 ppm on fifth instar, 24 h; LC_50_ = 81.81 on first instar, 7 days) Sargaquinoic acid (**7**) Mixture **1**, **3**, and **7** (6:3:1) (LD_50_ = 9.23 ppm on fifth instar, 24 h; LC_50_ = 17.76 on first instar, 7 days) Acetylated Mixture (LD_50_ = 3.26 ppm on fifth instar, 24 h; LC_50_ = 5.77 on first instar, 7 days)	[19]
	*Parthenium argentatum* * Asteraceae	AP	Argentatin A (**8**) (LD_50_ = 12.4 ppm on fifth instar, 24 h; LC_50_ = 17.8 ppm, 7 days; MC_50_ = 21.3 ppm, 7 days) Argentatin B (**9**) (LD_50_ = 19.8 ppm, on fifth instar, 24 h; LC_50_ = 36.1 ppm, 7 days; MC_50_ = 37 ppm, 7 days) Methanol (LD_50_ = 3.1 ppm, on fifth instar, 24 h; LC_50_ = 6.4 ppm, 7 days; MC_50_ = 6.9 ppm, 7 days)	[20]
	*Gutierreza microcephala* * Asteraceae	AP	Bacchabolivic acid (**10**) (MC_50_ = 10.7 ppm, 7 days; LD_50_ = 6.59 ppm, 24 h; 50 ppm: 90.2% IAche) Methyl ester of **10** (**10a**) (MC_50_ = 3.46 ppm, 7 days; LD_50_ = 15.05 ppm, 24 h; 50 ppm: 60% IAche)	[21]
	Cedrela dugessi * Meliaceae	Leaves	α and β-Photogedunin (**11** and **12**) mixture (LC_50_ = 10 ppm, 7days; 19.2 ppm: 88% larval growth inhibition; 5 ppm: 23 and 85% pupation and emergence reduction) α and β- Photogedunin acetates (**11a** and **12a**) mixture (LC_50_ = 8 ppm, 7 days) Gedunin (**13**) (LC_50_ = 39 ppm, 7days; 5 ppm: 91% larval growth inhibition; 5 ppm: 6.2 and 78.5% pupation and emergence reduction)	[22]
	*Cedrela salvadorensis*	Leaves	α- and β-Photogedunin (**11** and **12**), α- and β- photogedunin acetates (**11a** and **12a**) mixture gedunin (**13**)	[22]
	*Vitex hemsleyi* * Lamiaceae	Leaves Stem	Anticopalic acid (**14**) (EC_50_ = 90.6 ppm, L6 larvae)	[23]
	*Myrtillocactus geometrizans* * Cactaceae	Whole	Macdougallin (**15**) (LD_95_ = 285 ppm; 50 ppm: 97.2% M; 0% pupation; 0% emergence) Peniocerol (**16**) (LD_95_ = 125 ppm; 50 ppm: 97.2% M; 0% pupation; 0% emergence) mixture (4:6) **15** + **16** (LD_95_ = 135 ppm; 20 ppm: 97.2% M; 0% pupation; 0% emergence)	[24]
	*Crescentia alata* Bignoniaceae	Fruits	Fraction enriched with ningpogenin (**17**) (100 ppm: 80% larval mortality); fraction enriched with: β-sitosterol (**18**), stigmasterol (**19**) and 6β,7β,8α,10-tetra-*p*-hydroxybenzoyl-*cis*-2-oxabicycle[4.3.0]nonan-3-one (**20**) (100 ppm: 65% larval mortality)	[25,26]
	*Lippia graveolens* Verbenaceae	Leaves	Hexane (10–100 ppm: deformed adults), thymol (**21**, 70.6%), carvacrol (**22**, 22.8%)	[27]
*S. littoralis*	Salvia keerlii * Lamiaceae	AP	Kerlinolide (**23**) (AI_50_ = 67 ppm)	[28]
	Salvia lineata * Lamiaceae	AP	1(10)-Dehydrosalviarin (**24**, AI_50_ = 32 ppm)	[28]
	Salvia melissodora * Lamiaceae	AP	13,14-Dihydro-3,4 epoxy-melissodoric acid methyl ester acetate (**25**) (AI_50_ = 1 ppm) 2-β-acetoxy-7α-hydroxy-*neo*-clerodan-3,13-dien-18,19:16.15-diolide (**26**) (AI_50_ = 84 ppm)	[28]
	Salvia rhyacophila * Lamiaceae	AP	Salviarin (**27**) (AI_50_ = 81 ppm) 6β-Hydroxysalviarin (**28**) (AI_50_ = 24 ppm)	[28]
	Salvia semiatrata * Lamiaceae	AP	Semiatrin (**29**) (AI_50_ = 87 ppm)	[28]
	Senecio toluccanus * Asteraceae	Roots	Toluccanolide A (**30**) and toluccanolide A acetate (**30a**) (50 μg/cm^2^: 57 and 69.6% antifeedant effect, respectively)	[29]

* Endemic; AP: Aerial parts; AI_50_ = Median antifeedant index; EC_50_ = Effective antifeedant concentration; GD_50_ = Median Growth Dose; ID_50_ = Median Inhibitory Dose; LC_50_ = Median Lethal Concentration; LD_50_ = Median Lethal Dose; LV_50_ = Median Larval Viability; IAche: Inhibition of acetylcholinesterase; MC_50_ = Median Mortality Concentration.

**Table 2:** Insecticidal flavonoids from Mexican flora effective on *Spodoptera frugiperda*.

Species/Family	Plant Part	Compound (Toxicity)	Ref.
*Gutierreza microcephala* * Asteraceae	AP	5,7,2′-Trihydroxy-3,6,8,4′,5′-pentamethoxyflavone (**31**) (MC_50_ = 3.9 ppm, 7 days; LD_50_ = 36.65 ppm, 24 h; 50 ppm: 35.9% IAche) 5,7,4′-Trihydroxy-3,6,8-trimethoxyflavone (**32**) (50 ppm: 27.5% IAche) 5,7,2′,4′-Tetrahydroxy-3,6,8,5′-tetramethoxyflavone (**33**) (MC_50_ = 27.8 ppm, 7 days; 50 ppm: 27.5% IAche) 5,2-dihydroxy-3,6,7,8,4′,5′-hexamethoxyflavone (**34**) (50 ppm: 17.8% IAche)	[21]

* Endmic; IAche: Inhibition of acetylcholinesterase; LD_50_ = Median Lethal Dose; MC_50_ = Median Mortality Concentration.

**Table 3:** Stilbenes from Mexican flora active on *Spodoptera frugiperda*.

Species/Family	Plant Part	Compound (Toxicity)	Ref.
*Yucca periculosa* * Asparagaceae	Bark	Resveratrol (**36**) (LD_50_ = 24.1 ppm, 24 h; GI_50_ = 5.94 ppm, 21 days; LC_50_ = 6.4 ppm, 7 days) 4,4′-Dihydroxystilbene (**37**) (LD_50_ = 38 ppm, 24 h; GI_50_ = 9.24 ppm, 21 days; LC_50_ = 27.6 ppm, 7 days) 3,3′,5,5′-Tetrahydroxy-4-methoxystilbene (**38**) (LD_50_ = 10.1 ppm, 24 h; GI_50_ = 3.45 ppm, 21 days; LC_50_ = 5.4 ppm, 7 days)	[31]

* Endemic; GI_50_ = Median Growth inhibition; LD_50_ = Median Lethal Dose; LC_50_ = Median Lethal Concentration.

**Table 4:** A coumarin and a ketone active on *Spodoptera frugiperda*.

Species/Family	Plant Part	Compound (Toxicity)	Ref.
*Ruta graveolens* Rutaceae	Leaves	Psoralen (**39**) (1 mg/mL: 100% larval mortality) 2-Undecanone (**40**) (1 mg/mL: 50% larval mortality)	[30]

**Table 5:** Fatty acids with biological activity on *Spodoptera frugiperda*.

Species/Family	Plant Part	Compound (Toxicity)	Ref.
*Carica papaya* Caricaceae	Seeds	Palmitic acid (**41**) (LV_50_ = 989 ppm) Oleic acid (**42**) (LV_50_ = 1353.4 ppm) Powder in artificial diet (15%: 90% mortality, 72 h, all varieties)	[32]
*Ricinus communis* Euphorbiaceae	Leaves	Linoleic acid (**43**) (LV_50_ = 857 ppm, 1st instar larvae) Linolenic acid (**44**) (LV_50_ = 849 ppm, 1st instar larvae)	[33]

LV_50_ = Median Lethal Volume.

**Table 6:** Alkaloids effective on *Spodoptera frugiperda*.

Plant Species/Family	Plant Part	Compound (Toxicity)	Ref.
Lupinus aschenbornii * Fabaceae	Leaves	Alkaloids extract (LD_50_ = 24 μg/mL, 7 days) Lupanine (**45**, 86 μg/g), multiflorine (**46**, 31 μg/g), sparteine (**47**, 780 μg/g), **47** commercial standard (LD_50_ = 11 μg/mL, 7 days)	[37]
Lupinus montanus * Fabaceae	Leaves	Alkaloids extract (LD_50_ = 65 μg/mL, 7 days) Aphylline (**48**, 17.6 μg/g), **45** (9.2 μg/g), α-sparteine (**49**, 5 μg/g), **47** (640 μg/g)	[37]
Lupinus stipulates * Fabaceae	Seeds	Alkaloids extract (LD_50_ = 20 μg/mL, 7 days) **48** (280 μg/g), *epi*-aphylline-like (**50**, 307 μg/g), **45** (11.7 μg/g)	[37]

* Endemic; LD_50_ = Median Lethal Dose.

**Table 7:** Plant extracts from Mexican flora with activity on *Spodoptera* sp.

Insect	Plant Species/Family	Plant Part	Extract (Toxicity)	Ref.
*S. exigua*	*Trichilia havanensis* * Meliaceae	Seeds	Oil (7000 mg/L: 56% LM, 12 days; 100 mg/L: 71.3% LWR) Solid fraction (7000 mg/L: 56% LM, 12 days; 100 mg/L: 98.5% LWR)	[38]
*S. frugiperda*	*Bursera copallifera* * Burseraceae	Leaves	Ethyl acetate (1000 ppm: 73% LWR, 7 days; IC_50_ = 553 µg/mL IAche) Methanol (1000 ppm: 55% LWR, 7 days; IC_50_ = 367 µg/mL IAche)	[42]
		Leaves stem	Acetonic leaves extract (500 ppm: 47% LM; 50% LWR, 14 days); hexanic leaves extract (500 ppm: 44% deformed pupae, 14 days);	[41]
	*Bursera grandifolia* * Burseraceae	Leaves	Methanol leaves extract (500 ppm: 45% LM; 35% deformed pupae, 14 days)	[41]
	*Bursera lancifolia* * Burseraceae	Seeds	Ethyl acetate (1000 ppm: 39% LWR, 7 days; IC_50_ = 397 µg/mL IAche) Methanol (1000 ppm: 32% LWR, 7 days; IC_50_ = 707 µg/mL IAche)	[42]
	*Ipomoea murucoides* * Convolvulaceae	Roots	Methanol (LC_50_ = 2.69 mg/mL)	[45]
	*Ipomoea pauciflora* * Convolvulaceae	Seeds	Hexane (LC_50_ = 1.68 mg/mL) Chloroform (LC_50_ = 0.55 mg/mL)	[43]
	*Salvia connivens* * Lamiaceae	AP	Chloroform (LV_50_ = 936 ppm, 1st instar larvae)	[44]
	*Salvia microphylla* Lamiaceae	AP	Chloroform (LV_50_ = 916 ppm, 1st instar larvae)	[44]
	*Tagetes erecta* Asteraceae	Leaves	Hexane, acetone, and ethanol (LC_50_ = 312.2, 264.9, and 152.2 ppm respectively on L1 larvae)	[40]
	*Vitex mollis* * Lamiaceae	Leaves	Dichloromethane (LC_50_ = 46.35 ppm) Chloroform-methanol 1:1 (LC_50_ = 13.63 ppm) methanol (LC_50_ = 61.05 ppm)	[39]

* Endemic; IC_50_ = Median Inhibitory Concentration; IAche: Inhibition of acetylcholinesterase; LC_50_ = Median Lethal Concentration; LV_50_ = Median Larval Viability; LM: larval mortality; LWR: larval weight reduction.

**Table 8:** Metabolites from Mexican flora with effect against Culicidae.

Insect	Species/Family	Plant Part	Compound/Extract (Toxicity)	Ref.
*Aedes aegypti*	*Heliopsis longipes* * Asteraceae	Roots	Ethanol (LC_50_ = 4.07 mg/L, LM 48 h) Affinin (**51**) (LC_50_ = 7.38 mg/L, LM 48 h) *N*-Isobutyl-2*E*-decenamide (**52**) (LC_50_ = 36.97 mg/L, LM, 48 h)	[46]
	*Salmea scandens* * Asteraceae	Stem bark	EOs (LC_50_ = 0.3 μg/mL, 24 h) *N*-isobutyl-(2*E*,4*E*,8*Z*,10*Z*)-dodecatetraenamide (**55**, 22.5%) *N*-isobutyl-(2*E*,4*E*,8*Z*,10E)-dodecatetraenamide (**56**, 17.2%)	[48]
*Anopheles albimanus*	*Heliopsis longipes* * Asteraceae	Roots	Ethanol (LC_50_ = 2.48 mg/L, LM 48 h) **51** (LC_50_ = 4.24 mg/L, LM 48 h) **52** (LC_50_ = 7.47 mg/L, LM 48 h)	[46]
	*Salmea scandens* * Asteraceae	Stem bark	EOs (LC_50_ = 2.5 μg/mL, 24 h)	[48]
*Culex quinquefasciatus*	*Erythrina americana* Fabaceae	Seeds	Alkaloidal fraction (LC_50_ = 87.5 mg L^−1^, LM) β-eritroidina (**53**, LC_50_ = 225 mg L^−1^; LM) Erisovina (**54**, LC_50_ = 399 mg L^−1^, LM)	[47]
	*Persea Americana* Lauraceae	Leaves	EOs (50 mg/L: 40% mortality); (800 mg/L: 57.5% mortality; RGI = 0.74) estragole (**57**) (61.86%), sabinene (**58**, 15.16%), α-pinene (**59**, 14.25%)	[49]
	*Pseudocalymma alliaceum* * Bignonaceae	Fresh leaves	EOs: (LC_50_ = 385.29 ppm, 48 h) hydrolat (LC_50_ = 9.05%, 48 h) diallyl disulphide (**60**) (50.05%), diallyl sulphide (**61**, 11.77%), trisulphide di-2-propenyl (**62**, 10.37%)	[50]

* Endemic; Eos = Essential Oils; LC_50_ = Median Lethal Concentration; LM =Larval Mortality.

**Table 9:** Plant extracts from Mexican flora with activity on *Aedes aegypti* and *Culex quinquefasciatus*.

Insect	Species/Family	Plant Part	Extract (Toxicity)	Ref.
*Aedes aegypti*	*Argemone mexicana* Papaveraceae	Seeds	Hexane (LC_50_ = 80 μg /mL, 48 h) acetone (LC_50_ = 50 μg/mL, 48 h)	[51]
	*Pseudosmodingium perniciosum* * Anacardiaceae	Stem Bark	Hexane (LC_50_ = 20 μg/mL, 48 h)	[51]
	*Ruta chalepensis* Rutaceae	Aerial part	Ether and methanol (LC_50_ = 1.8 and 6.4 µg/mL, respectively, 24 h)	[52]
	*Thymus vulgaris* Lamiaceae	Leaves	Ether (LC_50_ = 4.4 ppm, 24 h, 4th instar larvae)	[52]
	*Zanthoxylum fagara* Rutaceae	Fruits	Ether (LC_50_ = 75.1 µg/mL, 24 h)	[52]
*Culex quinquefasciatus*	*Azadirachta indica* Meliacea	Seeds	Aqueous (1st instar: LD_50_ = 460 ppm; 2nd instar LD_50_ = 440 ppm; 3rd instar LD_50_ = 410 ppm; 4th instar; LD_50_ = 550 ppm)	[53]

* Endemic; LC_50_ = Median Lethal Concentration; LD_50_ = Median Lethal Dose.

**Table 10:** Plant extracts from Mexican flora with activity on *Anastrepha ludens* and *Bactericera cockerelli.*

Insect	Species/Family	Plant Part	Extract (Toxicity)	Ref.
*Anastrepha ludens*	*Annona diversifolia* Annonaceae	Leaves Stems	Ethanol stems (1000 μg/mL: 89.3%, third instar LM, 72 h) Aqueous leaves (100 μg/mL: 70.3% third instar LM, 72 h) Aqueous stems (1000 μg/mL: 74.3 third instar LM, 72 h)	[54]
	*Annona lutescens* Annonaceae	Leaves Stems	Ethanol leaves (100 μg/mL: 27.0%, third instar LM, 72 h) Ethanol stems (1000 μg/mL: 70.3%, third instar LM, 72 h) Aqueous leaves (100 μg/mL: 81.7% third instar LM, 72h) Aqueous stems (100 μg/mL: 95.9% third instar LM, 72 h)	[54]
	*Annona muricata* Annonaceae	Leaves Stems	Ethanol leaves (100 μg/mL: 63.3%, third instar LM, 72 h) Ethanol stems (1000 μg/mL: 61.3%, third instar LM, 72 h) Aqueous leaves (100 μg/mL: 78.3% third instar LM, 72 h) Aqueous stems (100 μg/mL: 86.0 third instar LM, 72 h)	[54]
	*Magnolia dealbata* Magnoliaceae	Dry sarcotesta	Ethanol (0.1 mg/mL: 12.8% survival after 3 days; Abbott index: 86.8%, adults)	[55]
*Bactericera cockerelli*	*Annona muricata* Annonaceae	Seeds	Hexanol (LC_50_ = 193.5 ppm, 72 h)	[55]

LC_50_ = Median Lethal Concentration LM: Larval Mortality.

**Table 11:** Plant extracts from Mexican flora with activity on *Bemisia tabaci.*

Species/Family	Plant Part	Extract (Toxicity)	Ref.
Acalypha gaumeri * Euphorbiaceae	Leaves	Aqueous (LC_50_ = 0.39% *w*/*v* on egg, 48 h) Ethanol (LC_50_ = 3.54 mg/mL on eggs; 3.15 mg/mL on nymphs, 48 h)	[57]
*Annona squamosa* Annonaceae	Leaves	Aqueous (LC_50_ = 0.36% *w*/*v* on eggs, 48 h) Ethanol (LC_50_ = 2.71 mg/mL on eggs, 48 h; 2.66 mg/mL on nymphs, 48 h)	[57]
*Agave tequilana* Asparagaceae	Leaves	Juice (undiluted: 31% mortality on adults) hexane (4%: 100% mortality on adults)	[58]
*Azadirachta indica* Meliacea	Leaves	Aqueous (LC_50_ = 0.30% *w*/*v* eggs, 48 h) Ethanol (LC_50_ = 4.14 mg/mL, eggs, 48 h; 10 ppm: 99.3% mortality of nymphs)	[57]
*Capsicum chinense* Solanaceae	Fruits	Ethanol (LC_50_ = 29.4% *w*/*v*; LT_50_ = 7.31 h; RI = 0.11)	[59]
*Carlowrightia myriantha* * Acanthaceae	Leaves	Aqueous (LC_50_ = 1.1% *w*/*v* on eggs) Ethanol (LC_50_ = 2.69 mg/mL on eggs; 3.10 mg/mL on nymphs)	[57]
*Chenopodium ambrosioides* Chenopodiaceae	Leaves Stems	Ethanol (LC_50_: 3.26% *w*/*v*, resuspended in water)	[60]
*Petiveria alliacea* Petiveriaceae	Aerial part	Aqueous (LC_50_ = 0.42% *w*/*v* on eggs) Ethanol (LC_50_ = 2.09 mg/mL on eggs; 1.27 mg/mL on nymphs)	[57]
*Piper nigrum* Piperaceae	Fruits	Ethanol (LC_50_: 1.6% *w*/*v*, resuspended in water)	[60]
*Pluchea serícea* Asteraceae	Leaves Stems	Aqueous leaves (LC_50_: 1190 ppm; RI = 0.52 on adults, 24 h) Acetone leaves (LC_50_: 700 ppm; RI = 0.78 on adults, 24 h) Ethanol leaves (LC_50_: 1250 ppm RI = 0.66 on adults, 24 h) Aqueous stems (LC_50_: 2620 ppm; RI = 0.54 on adults, 24 h)	[61]
*Trichilia arborea* Meliaceae	Leaves	Aqueous (LC_50_ = 0.39% *w*/*v* on eggs, 48 h) Ethanol (LC_50_ = 2.14 mg/mL on eggs, 48 h; 1.61 mg/mL on nymphs)	[57]

* Endemic; LC_50_: Median Lethal Concentration; RI: Repellency index.

**Table 12:** Plant extracts from Mexican flora with activity against *Copitarsia decolora* and *Dactylopius opuntiae.*

Insect	Species/Family	Plant Part	Extract (Toxicity)	Ref.
*Copitarsia decolora*	*Beta vulgaris* Chenopodiaceae	Stems Leaves	EOs (0.5%: 19% and 27% increased larval and pupal period length; 99% reduced fecundity and fertility)	[62]
	*Chenopodium berlandieri* subsp. *nuttalliae* Chenopodiaceae	Whole plant	EOs (0.5%: 22% and 38% increased larval and pupal period length; 94% and 85% reduced fecundity and fertility)	[62]
	*Chenopodium graveolens* Chenopodiaceae	Whole plant	EOs (0.5%: 19% and 28% reduced larval and pupal period length; 75% and 96% reduced fecundity and fertility)	[62]
*Dactylopius opuntiae*	*Cymbopogon winterianus* Poaceae	Leaves	EOs (LC_50_ = 6.6 mL/100 mL on 1st instar cochineal)	[63]
	*Lippia graveolens* Verbenaceae	Leaves	EOs (LC_50_ = 5.2 mL/100 mL on cochineal mobile juveniles)	[63]
	*Mentha spicata* Lamiaceae	Leaves	EOs (LC_50_ = 0.8 mL/100 mL solvent on cochineal mobile juveniles). Carvone (**63**, 61.03%) and limonene (**64**, 15.18%)	[63]
	*Ocimum basilicum* Lamiaceae	Leaves	EOs (LC_50_ = 2.4 mL/100 mL solvent on cochineal mobile juveniles)	[63]

LC_50_ = Median Lethal Concentration.

**Table 13:** Metabolites from Mexican flora with activity against *Leptinotarsa decemlineata.*

Insect	Species/Family	Plant Part	Extract/Compound (Toxicity)	Ref.
*Leptinotarsa decemlineata*	*Senecio toluccanus* * Asteraceae	Roots	6-Hydroxyeuryopsin (**65**) and acetyloxyeuropsin (**65a**) (50 μg/cm^2^: 85.5% antifeedant effect)	[29]

* Endemic; LC_50_ = Median Lethal Concentration.

**Table 14:** Essential oils from Mexican flora with activity on *Prostephanus truncates.*

Insect	Species/Family	Plant Part	Extract/Compound (Toxicity)	Ref.
*Prostephanus truncates*	*Lippia palmeri* Verbenaceae	Leaves	EOs (LC_50_ = 320.52 μL/L mortality, 24 h); carvacrol (**22**, 5.2%), **21** (58.9%) *p*-cimene (**66**, 21.8%)	[64]

LC_50_ = Median Lethal Concentration

**Table 15:** Plant extracts and metabolites from Mexican flora with activity against *Sitophilus zeamais.*

Species/Family	Plant Part	Extract/Compound (Toxicity)	Ref.
*Hippocratea excels* * Asteraceae	Root cortex	1% Pristimerin (**67**) (AAI = 89.2% and M = 16%, 5 days)	[65]
*Eupatorium glabratum* Asteraceae	Leaves	EOs (LC_50_ = 16 (females) and 20 μL/mL (males) after 1 week); LT_50_ = 53 (females) and 70 h (males); α-pinene (**59**, 29.5), α-phellandrene (**68**, 19.6%)	[66]
*Lippia palmeri* * Verbenaceae	Leaves	EOs (LC_50_ = 441.45 μL/L mortality, 48 h) *p*-cimene (**66**, 21.8%), **21** (58.9%)	[64]
*Aster subulatus* Asteraceae	Leaves	1% Leaves powder (M = 80.5%, 15 days)	[67]
*Bahia absinthifolia* Asteraceae	Leaves	1% powder (AE = 21.6%, 55 days)	[67]
*Chrysactinia mexicana* Asteraceae	Leaves Flower	1% Leaves powder (M = 80.5%, 15 days; AE = 0.0%, 55 days) 1% Flower powder (AE = 45.0%, 55 days)	[67]
*Erigeron longipes* Asteraceae	Flower	1% powder (M = 88.3%, 15 days)	[67]
*Heliopsis annua* Asteraceae	Leaves	1% powder (M = 80.6%, 15 days)	[67]
*Heterotheca inuloides* var. *rosei* * Asteraceae	Leaves Flower	1% Leaf powder (M = 87.7%, 15 days; AE = 0.0%, 55 days) 1% Flower powder (M = 87.7%, 15 days; AE = 45.0%, 55 days)	[67]
*Hippocratea celastroides* Asteraceae	Roots	1% Dichloromethane (AAI = 70.7%, 5 days) 1% Hexane (AAI = 67.8%, 5 days) 1% Acetone (soluble part: AAI = 72.3%, precipitate: AAI = 73.9%, 5 days)	[65]
*Senecio flaccidus* Asteraceae	Flower	1% Powder (M = 80.7%, 55 days)	[67]
*Stevia serrata* Asteraceae	Leaves Flower	1% Leaf powder (M = 80.2%, 55 days) 1% Flower powder (M = 81.8%, 55 days)	[67]
*Zaluzania peruviana* Asteraceae	Leaves Flower	1% Leafs powder (M = 88.1%, 15 days; AE = 50.0%, 55 days) 1% Flower powder (M = 48.3%, 15 days; AE 40%, 55 days)	[67]
*Stauranthus perforates* Rutaceae	Roots	Powder mixed with maize kernels (1–3%: 91, 95.5. and 100% mortality respectively, 15 days)	[67]

* Endemic; AAI: Antifeedant Activity Index; AE: Adults emergence; M= Mortality; LC_50_ = Median Lethal Concentration; LT_50_ = Median Lethal Time.

**Table 16:** Plant extracts and a metabolite from Mexican flora with activity on *Stomoxys calcitrans* and *Scyphophorus acupunctatus*.

Insect	Species/Family	Plant Part	Extract/Compound (Toxicity)	Ref.
*Stomoxys calcitrans*	*Teloxys graveolens* Chenopodiaceae	Aerial part	Pinocembrine (**69**) (LC_50_ = 418.69 μg/mL, 3rd stage larvae, 24 h)	[69]
*Scyphophorus acupunctatus*	*Annona cherimola* Annonaceae	Seeds	Podwer (15% in artificial diet: 63% LM; larval, pupal, and adult weight reductions of 98.5, 40.6, and 45.0%, respectively, 24 days)	[70]
	*Carica papaya* Caricaceae	seeds	fresh seed (15% in artificial diet: 90% LM, 24 days) dry seed powder (15% in artificial diet: 100% LM, 24 days)	[70]
	*Trichilia havanensis* Meliacea	seeds	Seed powder (15% in artificial diet: 100% LM, 24 days)	[70]

LC_50_: Median Lethal Concentration; LM: Larval mortality.

**Table 17:** Plant extracts and metabolites from Mexican flora with activity on *Tenebrio molitor* and *Trichoplusia ni*.

Insect	Species/Family	Plant Part	Extract/Compound (Toxicity)	Ref.
*Tenebrio molitor*	*Myrtillocactus geometrizans* * Cactaceae	Whole plant	Macdougallin (**15**) (100 ppm: 5% survival) Peniocerol (**16**) (100 ppm: 3% survival) mixture (6:4) **15** + **16** (100 ppm: 0% survival)	[24]
*Trichoplusia ni*	*Azadirachta indica* Meliacea	Leaves	Volatile compounds released (1 and 10 g: 24% and 63% neonate mortality; 77% and 79% larval mortality; LD_50_ = 5.6 g, 7 days)	[71]

* Endemic; LD_50_: Median Lethal Dose.

**Table 18:** Plant extracts from Mexican flora with activity on *Trialeurodes vaporariorum*.

Species/Family	Plant Part	Extract (Toxicity)	Ref.
*Tagetes filifolia* Asteraceae	Flower Leaves Whole plant	Flower (RC_50_ = 0.13 mg/mL; LC_50_ = 6.59 mg/mL, 24 h; OIC_50_: 8.43 mg/mL, adults) Leaves (0.23 mg/mL; LC_50_ = 10.29 mg/mL, 24 h; OIC_50_: 3.88 mg/mL, adults) Whole plant (RC_50_ = 0.24 mg/mL; LC_50_ = 9.9 mg/mL, 24 h; OIC_50_: 3.56 mg/mL, adults) *trans*-anethole (**70**) commercial standard (RC_50_ = 0.45 mg/mL; LC_50_ = 1.74 mg/mL, 24 h; OIC_50_: 1.55 mg/mL, adults)	[72]
*Piper auritum* Piperaceae	Leaves stems	Ethanol (LC_50_ = 116 mg/mL on adult, 24 h) Acetone (IOC_50_ = 89.1 mg/mL on adult, 24 h)	[73]
*Raphanus raphanistrum* Brassicaceae	Leaves	Water (IOC_50_ = 77.3 mg/mL, on adult, 24 h) Ethanol (LC_50_ = 185.2 mg/mL, on adult, 24 h)	[73]
*Petiveria alliacea* Petiveriaceae	Aerial part	Laboratory assays: Aqueous (LC_50_ = 4.6%), methanol (LC_50_ = 1.1%), dichloromethane (LC_50_ = 0.3%), In greenhouse (tomato) aqueous (LC_50_ = 16.6%), methanol (LC_50_ = 13.3%), dichloromethane (LC_50_ = 3.5%)	[74]
*Arundo donax* Poaceae	Roots	Aqueous (non-active) Methanol (LC_50_ = 0.57% and 34.79% *w*/*v*, *in vitro* and greenhouse RC_50_ =, respectively)	[75]
*Phytolacca icosandra* Phytolaccaceae	Leaves stems	Aqueous (non-active) Methanol (LC_50_ = 0.34% and 36.47% *w*/*v*, *in vitro* and greenhouse, respectively) Qualitative analysis: Terpenoids and saponins	[75]

IOC_50_: Median Inhibition of Oviposition Concentration; LC_50_ = Median Lethal Concentration; RC_50_ = Median Repellent Concentration.

**Table 19:** Plant extracts from Mexican flora with insecticidal activity against *Zabrotes subfasciatus.*

Species/Family	Plant Part	Extract (Toxicity)	Ref.
*Senecio salignus* Asteraceae	Roots	Powder (male: LC_50_ = 0.03%, 3–6 days; LT_50_ = 1.31 days) (female: 0.08% 3–6 days; LT_50_ = 3.2 days)	[76]
*Lippia palmeri* * Verbenaceae	Leaves	EOs Puerto del oregano (LC_50_ = 1.35 μL/g mortality, 48), **22** (37.35%), **21** (24.56%), **64** (15.62%) Alamos (LC_50_ = 1.35 μL/g mortality, 48), **64** (33.70%), **22** (18.32%)	[77]

* Endemic; LC_50_: Median Lethal Concentration; LT_50_: Median Lethal Time.

**Table 20:** Phytonematicidal metabolites and plant extracts from Mexican flora.

Nematode	Species/Family	Plant Part	Compound/Extract (Toxicity)	Ref.
*Meloidogyne javanica*	*Lippia graveolens* Verbenaceae	Leaves	Hexane (LC_50_ = 0.672 mg/mL) **21** (70.6%), **22** (22.8%)	[27]
	*Sicyos bulbosus* * Cucurbitaceae	Roots	Tacacoside B3 (**71**) (0.5 µg/µL: 93% J_2_ I) tacacoside C (**72**) (0.5 µg/µL: 97% J_2_ I) 16-OH-tacacoside B3 (**73**) (0.5 µg/µL: 100% J_2_ I), durantanin III (**74**) (0.5 µg/µL: 74% J_2_ I) heteropappussaponin 7 rhamnoside (**75**) (0.5 µg/µL: 80% J_2_ I), heteropappussaponin 5 (**76**) (0.5 µg/µL: 91% J_2_ I) heteropappussaponin 7 (**77**) (0.5 µg/µL: 93% J_2_ I)	[78]
	*Microsechium helleri* * Cucurbitaceae	Roots	Amole F (**78**) (0.5 µg/µL: 4.78% J_2_ I) amole G (**79**) (0.5 µg/µL: 7.83% J_2_ I) 16-OH-amole F (**80**) (0.5 µg/µL: 6.52% J_2_ I) 16-OH-amole G (**81**) 0.5 µg/µL: 6.34% J_2_ I)	[78]
*Nacobbus aberrans*	*Capsicum annuum* Solanaceae	Roots	Capsidiol (**82**) (1 μg/mL: >80% J_2_ I, 72 h)	[79]
*Meloidogyne incognita*	*Calea urticifolia* Asteraceae	Roots	Ethanol (250 ppm: 80% larval mortality, 72 h) In greenhouse: Water (50% *w*/*v*: 72% decrease eggs formation; 50% galling reduction)	[80]
	Eugenia winzerlingii * Myrtaceae	Leaves	Ethanol (ED_50_ = 133.4 ppm)	[81]
	*Tephrosia cinerea* Fabaceae	Stem	Ethanol (250 ppm: 85% larval mortality, 72 h)	[81]

* Endemic; ED_50_: Median Effective Dose; I: Immobility; LC_50_: Median Lethal Concentration.

**Table 21:** Nematicidal metabolites and plant extracts from Mexican plants with activity on *Ascaridia galli*, *Cooperia puntacta*, and *Cyathostomin* sp.

Nematode	Species/Family	Plant Part	Compound/Extract (Toxicity)	Ref.
*Ascaridia galli*	*Teloxys graveolens* Chenopodiaceae	Aerial part	Pinocembrine (**69**) (LC_50_ = 623.49 μg/mL)	[69]
*Cooperia punctata*	*Leucaena leucocephala* Fabaceae	Fresh Leaves	Water (LC_50_ = 7.93 mg/mL EHI) Fraction LlC1F3 (LC_50_ = 0.06 mg/mL EHI) Quercetin (**83**, 82.21%), caffeic acid (**84**, 13.42%)	[82,83]
	*Gliricidia sepium* Fabaceae	Fresh Leaves	Acetone (LC_50_ = 1.03 mg/mL EHI) 2H-Chromen-2-one (**85**) (EC_50_ = 0.024 mg/mL EHI)	[84]
			Oxytroside (**86**) (2400 µg/mL inhibited exsheathment)	[85]
*Cyathostomin* sp.	*Diospyros anisandra* Ebenaceae	Leaves Bark	Methanol bark (LC_50_ = 10.28 µg/mL EHI in rainy season) Methanol leaves (LC_50_ = 18.48 µg/mL EHI in rainy season)	[86]
	*Petiveria alliacea* Petraceae	Stem	Methanol (LC_50_ = 28.27 µg/mL EHI in rainy season)	[86]

EC_50_: Median Effective Concentration; LC_50_: Median Lethal Concentration. EHI: Egg Hatching inhibition.

**Table 22:** Plant extracts and metabolites from Mexican flora with *in vitro* activity against *Haemonchus contortus.*

Species/Family	Plant Part	Extract (Toxicity)	Ref.
*Caesalpinia coriaria* Fabaceae	Fruits Leaves	Hydroalcoholic (fruits: LC_50_ = 1.63 mg/mL; leaves: LC_50_ = 3.98 mg/mL on EHI, 48 h)	[87]
*Phytolacca icosandra* Phytolaccaceae	Leaves	Dichloromethane (LD_50_ = 0.90 mg/mL LMI; LD_50_ = 0.28 mg/mL EHI) Ethanol (2 mg/mL: 55.4% LMI; 1.8 mg/mL: 95% EHI)	[88]
*Gliricidia sepium* Fabaceae	Leaves	Methanol (ED_50_ = 394.96 µg/mL EHI)	[89]
*Acacia cochliacantha* Fabaceae	Fresh Leaves	Hydroalcoholic (100 mg/mL: 100% EHI) Ethyl acetate (LC_50_ = 0.33 mg/mL EHI) Dichloromethane soluble fraction (LC_50_ = 0.06 mg/mL EHI) Dichloromethane precipitate (LC_50_ = 0.04 mg/mL EHI)	[90]
*Carica papaya* Caricaceae	Seeds	Ethanol (2.5 mg/mL: 92% EHI) Hydroalcoholic (2.5 mg/mL: 50% EHI)	[91]
*Acacia pennatula* Fabaceae	Leaves	Tannins (1200 µg/mL: 51% LMI)	[92]
*Arachis pintoi* Fabaceae	Leaves	Condensed tannins (4.5–45 µg/mL: 76.6–100% LM, 96 h)	[92]
*Guazuma ulmifolia* Malvaceae	Leaves	Condensed tannins (4.5–45 µg/mL: 86.0–99.4% LM, 96 h)	[92]
*Manihot esculenta* Euphorbiaceae	Leaves	Condensed tannins (4.5–45 µg/mL: 69.9–100%, LM, 96 h)	[92]
*Leucaena leucocephala* Fabaceae	Leaves	Condensed tannins (4.5–45 µg/mL: 71.0–98.4% LM, 96 h)	[92]
	Leaves	Tannin (1200 µg/mL: 53.6% LMI)	[93]
*Lysiloma latisiliquum* Fabaceae	Leaves Leaves	Tannin (1200 µg/mL: 49.1% LMI)	[93]
*Piscidia piscipula* Fabaceae	Leaves	Tannin (1200 µg/mL: 63.8% LMI)	[93]
*Laguncularia racemosa* Combretaceae	Leaves	30% Acetone–water (3600 µg/mL: 50.29 larvae failing eclosion)	[94]
*Senegalia gaumeri* * Fabaceae	Leaves	Acetona–water 70:30 (EC_50_= 401.8 EHI; 83.1 LMI)	[95]
*Bursera copallifera* * Burseraceae	Stem	Acetone (20 mg/mL: 66% LM, 72 h)	[96]
*Prosopis laevigata* Fabaceae	Aerial part	Hexane (20 mg/mL: 86% LM, 72 h postexposure)	[96]
*Cydista aequinoctialis* Bignonaceae	Leaves	Aqueous (20 mg/mL: 39.57% LM, 72 h)	[97]
*Heliotropium indicum* * Boraginaceae	Leaves	Aqueous (20 mg/mL: 34.59% LM, 48 h)	[97]
*Momordica charantia* Cucurbitaceae	Leaves Fruits	Aqueous (20 mg/mL: 53.83% LM, 72 h) Aqueous (20 mg/mL: 68.13% LM, 72 h)	[97]
*Larrea tridentata* Zygophyllaceae	Leaves	Hydro-methanol 30% (EC_50_ = 36 mg/mL on exsheathed larvae, 24 h)	[98]
*Allium sativum* Amaryllidaceae	Bulbs	Hexane (LC_50_ = 3.8 mg/mL LM, 72 h)	[99]
*Tagetes erecta* Asteraceae	Flowers	Acetone (40 mg/mL: 36.6% LM, 72 h)	[99]
*A. sativum-T. erecta*	Combined	Combined bulbs and flower (LC_50_ = 1.3 mg/mL LM, 72 h)	[99]
*Castela tortuosa* * Simaroubaceae	Aerial part	Hexane (LC_50_ = 17.3 mg/mL EGI, 72 h)	[100]
*Chenopodium ambrosioides* Chenopodiaceae	Aerial part	Hexane (LC_50_ = 1.5 mg/mL EGI, 72 h)	[100]
*C. ambrosioides*- *C. tortuosa*	Combined	Hexane (LC_50_ = 6.5 mg/mL EGI, 72 h)	[100]

* Endemic; EHI: Egg hatch inhibition; LM: Larval mortality; LMI: larval migration inhibition; LC_50_: Median Lethal Concentration; LD_50_: Median Lethal Dose.

**Table 23:** The *in vivo* evaluations of plant extracts against *Haemonchus contortus.*

Plant Species	Host	Sample (Toxicity)	Ref.
*Allium sativum* Amaryllidaceae	Gerbils	Oral administration extract (40 mg/mL) (100 µL: 68.7% LPR)	[99]
*Tagetes erecta* Asteraceae	Gerbils	Oral administration extract (40 mg/mL) (100 µL: 53.9% LPR)	[99]
*Allium sativum-Tagetes erecta* 1:1 combined	Gerbils	Oral administration combined extract (40 mg/mL) (100 µL: 87.5% LPR)	[97]
*Castela tortuosa* *	Gerbils	Hexane extract intraperitoneally administred (40 mg/kg BW: 27.15% LPR)	[100]
*Chenopodium ambrosioides*	Gerbils	Hexane extract (100 µL) intraperitoneally administred (40 mg/kg: 45.86% LPR)	[100]
*Castela tortuosa Chenopodium ambrosioides* combined	Gerbils	Hexane extract (100 µL) intraperitoneally administred (40 mg/kg BW: 57.36% LPR)	[100]
*Prosopis laevigata*	Gerbils	Hexane extract (40 mg/mL) intraperitoneally administred (100 µL: 42.5% reduced the parasite population)	[101]
*Lysiloma acapulcensis* *	Lambs	Ethyl acetate fraction (25 mg/kg BW: 94.8% EHI; 62.9% EPGR) Dried leaves (5g/kg BW: 50.1% EPGR)	[102]
	Sheep	Rutin (**36**) (10 mg/kg BW: 66.2% EPGR)	[102]
*Phytolacca icosandra*	Sheep	Ethanol (250 mg/kg, 2 days: 72% reduction on eggs/g of faeces)	[103]
*Oxalis tetraphylla* Oxalidaceae	Lambs	(20 mg/kg: 45.6% reduction in the eggs/g of feces)	[104]
*Acacia cochliacantha*	Goats	Fresh foliage (1.48 log^10^ excreted eggs per gram; control 2.18 log^10^; 0.6 kg/ animal weight gained)	[105]
*Pithecellobium dulce*	Goats	Fresh foliage (1.18 log^10^ excreted eggs per gram; control 2.18 log^10^; 2.4 kg/ animal weight gained)	[105]

* Endemic; BW: Body weight; EHI: Egg hatch inhibition; EPGR: Egg per gram reduction; LPR: Larval population reduction.

**Table 24:** Extracts from Mexican plants active on *Trichostrongylus colubriformis.*

Species/Family	Plant Part	Extract (Toxicity)	Ref.
*Acacia pennatula* Fabaceae	Leaves	Tannin (1200 µg/mL: 71% Lm)	[106]
*Leucaena leucocephala* Fabaceae	Leaves	Tannin (1200 µg/mL: 72% Lm)	[106]
*Lysiloma latisiliquum* Fabaceae	Leaves	Tannin (1200 µg/mL: 56% Lm)	[106]

Lm: larval migration of third-stage larvae.
